# Understanding blaNDM-1 gene regulation in CRKP infections: toward novel antimicrobial strategies for hospital-acquired pneumonia

**DOI:** 10.1186/s10020-024-00794-y

**Published:** 2024-02-23

**Authors:** Liang Ding, Zheng Yang, Baier Sun

**Affiliations:** Department of Respiratory and Critical Care Medicine, Affiliated Hospital of Nantong University, No. 20, Xisi Road, Chongchuan District, Nantong, 226001 Jiangsu Province China

**Keywords:** Hospital-acquired pneumonia, CRKP, blaNDM-1 gene, Antimicrobial strategy, Gene expression regulation, Drug resistance

## Abstract

**Background:**

The escalating challenge of Carbapenem-resistant *Klebsiella pneumoniae* (CRKP) in hospital-acquired pneumonia (HAP) is closely linked to the blaNDM-1 gene. This study explores the regulatory mechanisms of blaNDM-1 expression and aims to enhance antibacterial tactics to counteract the spread and infection of resistant bacteria.

**Methods:**

KP and CRKP strains were isolated from HAP patients' blood samples. Transcriptomic sequencing (RNA-seq) identified significant upregulation of blaNDM-1 gene expression in CRKP strains. Bioinformatics analysis revealed blaNDM-1 gene involvement in beta-lactam resistance pathways. CRISPR-Cas9 was used to delete the blaNDM-1 gene, restoring sensitivity. In vitro and in vivo experiments demonstrated enhanced efficacy with Imipenem and Thanatin or Subatan combination therapy.

**Results:**

KP and CRKP strains were isolated with significant upregulation of blaNDM-1 in CRKP strains identified by RNA-seq. The Beta-lactam resistance pathway was implicated in bioinformatics analysis. Knockout of blaNDM-1 reinstated sensitivity in CRKP strains. Further, co-treatment with Imipenem, Thanatin, or Subactam markedly improved antimicrobial effectiveness.

**Conclusion:**

Silencing blaNDM-1 in CRKP strains from HAP patients weakens their Carbapenem resistance and optimizes antibacterial strategies. These results provide new theoretical insights and practical methods for treating resistant bacterial infections.

**Supplementary Information:**

The online version contains supplementary material available at 10.1186/s10020-024-00794-y.

## Introduction

Pneumonia is a major contributor to global mortality, and hospital-acquired pneumonia (HAP) refers explicitly to lung infections contracted by patients in healthcare settings, usually within 48 h of admission. HAP risk factors comprise the patient's immune status, mechanical ventilation, surgery, and antibiotic usage (Modi and Kovacs 2020; Bassetti et al. 2022; Wicky et al. 2022). HAP not only lengthens patients' hospital stays and increases the costs of treatment, but it could also result in severe complications and even death (Lanks et al. 2019; Suaya et al. 2021; Yusuf et al. 2021). *Klebsiella pneumoniae* (KP) is a gram-negative opportunistic pathogen belonging to Klebsiella, mainly found in the gastrointestinal tract and nasopharynx (Bassetti et al. 2018a; Bassetti et al. 2018b). KP invades the bloodstream via the nasal and pharyngeal regions and is recognized as a predominant pathogenic bacterium responsible for HAP globally (Li et al. 2023a).

In recent years, the widespread and excessive use of antibiotics in clinical settings has led to increasing antibiotic-resistant strains of KP with continuously strengthening resistance. It has presented a challenge for clinical treatment. Approximately 700,000 deaths per year are attributed to antibiotic resistance, and projections suggest that by 2050, antibiotic resistance will become the most catastrophic issue, resulting in an annual death toll of up to 10 million (Bassetti et al. 2018a; Han et al. 2022). Currently, carbapenems are the most comprehensive and potent class of antibacterial drugs in clinical practice. Historically, they have proven to be the most effective treatment for KP infections. As a result, carbapenems are frequently regarded as the final resort for combating multidrug-resistant bacterial infections in clinical settings (Mora-Ochomogo and Lohans 2021; Yang et al. 2021a; Lan et al. 2021; Liao et al. 2020).

The high rates of infection and mortality caused by CRKP strains have burdened the national healthcare system. Moreover, the growing bacterial resistance has exerted immense pressure on developing new drugs (Xu et al. 2017). Currently, the clinical treatment of KP faces numerous challenges and controversies, necessitating the development of more effective methods (Bassetti et al. 2018a; Isler et al. 2022). The Metallo-β-lactamase, encoded by the blaNDM-1 gene, is critical in developing multidrug-resistant strains in these bacteria. However, there is limited knowledge regarding regulating blaNDM-1 gene expression and its connection to drug resistance (Bahr et al. 2021).

This study aims to address the issue by investigating the regulatory mechanism of blaNDM-1 gene expression in CRKP strains among HAP patients and identifying methods to optimize antimicrobial strategies. Transcriptome sequencing could provide a comprehensive insight into the expression differences of the blaNDM-1 gene in resistant and susceptible strains, thus enabling exploration of the molecular basis underlying their resistance.

Moreover, we utilized bioinformatics analysis tools to analyze the RNA-seq data further. This analysis aims to identify the signaling pathways and regulatory networks associated with drug resistance and involvement in the blaNDM-1 gene. Additional investigation was carried out to examine the effect of disrupting the blaNDM-1 Gene on the bacterial strain's drug resistance in laboratory and animal models.

Finally, we conducted in vitro and in vivo experiments to evaluate the efficacy of the optimized antibacterial strategy targeting the blaNDM-1 gene. Studies have shown that the antimicrobial peptide Thanatin (TH) disrupts the outer membrane of NDM-1-producing bacteria by competitively replacing divalent cations and inducing lipopolysaccharide release. Moreover, Thanatin inhibits NDM-1 enzyme activity by competitively replacing zinc ions at the active site, thereby reversing carbapenem resistance in NDM-1-producing bacteria in vitro and in vivo (Ma et al. 2019). Based on these findings, we have selected to combine the carbapenem antibiotic imipenem (IM) with TH as a therapeutic approach against CRKP. Additionally, literature reports have indicated that sulbactam (SU), an irreversible competitive β-lactamase inhibitor, prevents the degradation of β-lactam antibiotics by inhibiting the activity of β-lactamases (Noguchi and Gill 1988). Therefore, we have chosen to combine SU with the carbapenem antibiotic IM to investigate the therapeutic effect of combination therapy against CRKP strain infections. Through the aforementioned research, we aim to provide new insights into the resistance mechanisms of CRKP strains, especially the expression regulation of the blaNDM-1 gene, to offer more effective strategies for clinical treatment, address the threat of antibiotic resistance, and promote advancements in the field of public health.

The current study addresses the knowledge gap in regulating the blaNDM-1 gene and proposes novel therapeutic strategies for CRKP-induced infections. As the issue of antibiotic resistance becomes more severe, this analysis aims to provide robust support for developing new antimicrobial drugs and treatment strategies. It also seeks solutions for the challenges posed by antimicrobial resistance in clinical treatment, ultimately improving patients' cure and survival rates with infectious diseases. Furthermore, acquiring a comprehensive knowledge of blaNDM1 gene regulation and its associated resistance mechanisms will establish a novel theoretical framework for tailor-made design and precise medication in antibacterial therapy. Additionally, it will offer scientific substantiation for the development and execution of antimicrobial strategies in the public health sphere.

## Materials and methods

### Declaration of clinical research ethics

Informed consent was obtained from all patients whose clinical samples were involved in this investigation. The sample collection and processing procedures strictly followed ethical guidelines. All participants have been provided with clear information about the research purpose and have willingly signed consent forms that outline the details of the study. This study has received approval from the Ethics Committee of the Affiliated Hospital of Nantong University (No. 2020-K044) and strictly adhered to the Helsinki Declaration.

### Declaration of animal research ethics

All animal experiments in our study adhered to the regulations and guidelines established by the Animal Experiment Ethics Committee of the Affiliated Hospital of Nantong University (No. 2020-K044) and received necessary approvals. Every experiment aims to minimize the pain and discomfort experienced by animals and reduce the number of animals needed for testing to the fullest extent possible.

### Inclusion criteria for HAP patients

Based on the clinical and pathogenic diagnostic criteria for HAP, one patient treated with carbapenem antibiotics and another untreated with carbapenem antibiotics for HAP were selected. From the blood samples of these two HAP patients, strains of CRKP and KP were isolated.

The clinical diagnostic criteria for HAP were as follows: (1) The condition was neither present at the time of hospital admission nor in the incubation period of any pathogen. Instead, it developed more than 48 h after admission. This condition encompassed ventilator-associated pneumonia, which occurred in patients who underwent tracheal intubation or tracheostomy and received mechanical ventilation for at least 48 h during hospitalization and those who developed symptoms within 48 h post-extubation or cessation of mechanical ventilation; (2) Chest X-ray or CT scans revealed new or progressive infiltrates, consolidations, or ground-glass opacities; (3) The patients exhibited fever with body temperatures exceeding 38 ℃; (4) Purulent secretions were present in the respiratory tract; (5) Peripheral white blood cell counts were either above 10 × 10^9^/L or below 4 × 10^9^/L. A diagnosis of HAP was established when a patient met the criteria of (1) and (2), along with at least two of the criteria from (3) to (5).

The pathogenic diagnosis criteria for HAP included: (1) Qualified lower respiratory tract secretions: more than 25 neutrophils per low power field, fewer than 10 epithelial cells per low power field, or a ratio exceeding 2.5:1; (2) Bacteria cultured from bronchoscopic protected brush samples, bronchoalveolar lavage fluid, lung tissue, or sterile body fluid, with clinical manifestations corresponding to the cultured bacteria.

### Isolation and culture of KP

The blood was centrifuged at 500 rpm for 5 min to remove blood cell sediment, and the resulting supernatant was collected. The obtained supernatant was then centrifuged at 4000 rpm for 5 min to obtain bacterial sediment. This sediment was streaked onto solid Luria–Bertani (LB) agar plates (PM0020P, Beijing Coolabo Technology Co., Ltd.) and incubated overnight at 37 ℃. Using an inoculation loop, colonies showing a gray-white, moist, smooth, and well-defined mucoid appearance, which were quickly streaked, were selected for further cultivation. These steps were repeated until colonies of uniform size emerged. To initiate bacterial amplification, select a single bacterial colony. Streak the log phase bacteria onto a MacConkey agar plate (HBPM016, Qingdao High-Tech Industrial Park Haibo Biotechnology Co., Ltd.) and incubate the plate overnight at 37 °C in a constant temperature incubator. Subsequently, reassess the colony's size, morphology, color, dryness, and viscosity (Kim et al. 2002).

### Characterization of KP

First, the bacterial strains were subjected to Gram staining using the Gram staining kit (SL7040, Beijing Coolabo Technology Co., Ltd., Beijing, China). The procedure was carried out strictly following the instructions provided in the manual. After drying, observations were made using an oil immersion lens.

The following method was used to detect the high viscosity phenotype. The strain was cultured overnight on an agar plate at 37 °C. Subsequently, an inoculation loop stretched the colonies on the same agar plate. Mucus was considered to have high viscosity if its length exceeds 5 mm.

The protocol for biochemical experiments is as follows: Purified bacteria were seeded into an LB liquid culture medium (PM0010L, Coolab Technology Co., Ltd., Beijing, China) and incubated for 24 h. Biochemical tests were then performed on the isolated strains using urease, glucoside, lactose, glucose, sucrose, maltose, lysine decarboxylase, ornithine decarboxylase, and hydrogen sulfide test tubes (A039, A040, A015, A014, A019, A016, A061, A060, A066) obtained from Hangzhou Binhe Microbial Reagent Co., Ltd., located in Hangzhou, China.

The operational procedure follows: LB liquid medium containing bacteria was taken and then inoculated into each microbiochemical reaction tube. The tubes were incubated at 35 ℃ for 24 h, and the reaction results were observed. In the urease test, the positive reaction solution turned red, while the negative reaction solution turned yellow. In the esculin test, the positive reaction solution turns dark brown, whereas the adverse reaction solution remains unchanged. The positive reaction solution turned yellow in the lactose test, while the adverse reaction solution remained unchanged. The positive reaction solution turns yellow in the glucose test, whereas the negative solution remains unchanged. In the sucrose test, the positive reaction solution turns yellow, while the negative reaction solution remains unchanged. The positive reaction solution turns yellow in the maltose test, whereas the negative reaction solution remains unchanged. In the lysine decarboxylase test, the positive reaction solution turned purple-red, whereas the negative reaction solution turned yellow. In the ornithine decarboxylase test, the positive reaction solution turned purple-red, while the negative reaction solution turned yellow. The positive reaction solution turned black in the hydrogen sulfide test, while the negative reaction solution did not.

The method for the Glucose Phosphate Protein Peptone Water Biochemical Test (V-P Test) is as follows: The bacterial sample is directly inoculated into VP culture tubes (J2072, Hangzhou Microbiology Reagent Co., Ltd., China) and incubated at 35 °C for 24 h. A positive result is indicated by the occurrence of a red color reaction, whereas a negative result is indicated by the reaction solution remaining colorless.

The indigo matrix biochemical test method is performed as follows: a small amount of pure culture of the tested bacteria is inoculated into a test culture medium tube. After 24 h of incubation at 37 °C, approximately 2 mL of culture fluid is collected. Then, 2–3 drops of indigo matrix reagent (D002, Hangzhou, China, Hangzhou Binho Microbial Reagent Co., Ltd.) are added to the tube, and the contents are gently shaken to mix the reaction solution thoroughly. A positive reaction is indicated by the color red, whereas the negative reaction solution does not change in color (Garza-Ramos et al. 2016).

### Extraction of bacterial genomes and PCR amplification of 16S rRNA genes

Bacterial genomic DNA extraction was performed: A purified single colony was placed into distilled water, heated to boiling, then cooled to room temperature. The mixture was centrifuged, and the supernatant was collected as the PCR template. The primer sequences utilized for the amplification of the 16S rRNA gene were as follows: Forward primer sequence: 5ʹ-AGAGTTTGATCCTGGCTCAG-3ʹ and Reverse primer sequence: 5ʹ-GGTTACCTTGTTACGACTT-3ʹ. The anticipated length of the PCR product is 1500 base pairs. Sangon Biotech (Shanghai) Co., Ltd. synthesizes the primers. The amplification of the 16S rRNA gene was detected using the PCR agarose gel electrophoresis method.

The PCR amplification system (50 μL) consisted of 25 μL 2 × Taq PCR Master Mix, 1 μL of each forward and reverse primer, 2 μL of DNA template, and the volume was adjusted to 50 μL with sterile water. The PCR reaction conditions were meticulously set according to the instructions provided in the manual. The PCR products were visualized using a fluorescence quantitative PCR instrument (Bio-Rad).

Sequencing and constructing the phylogenetic tree: The PCR products were sent to Sangon Biotech (Shanghai) Co., Ltd. for sequencing. The NCBI Blast tool was employed to compare and analyze the sequencing results with known related nucleotide sequences in GenBank. Subsequently, the Neighbor-joining method in the MEGA11 software was utilized to construct a phylogenetic tree (Uqab et al. 2022; Tamura et al. 2021).

### Drug sensitivity test

As per the instruction provided by the antimicrobial susceptibility testing paper, we conducted a drug susceptibility analysis on the isolated strains. The tested drugs included Ertapenem, IM, Meropenem, Chloramphenicol, Ciprofloxacin, Levofloxacin, Ceftazidime, Ceftriaxone, Cefotaxime, and Amikacin (item numbers: Z51080, Z51049, Z51050, Z51032, Z51034, Z51015, Z51014, Z51004, Z51002, Z51007, and Z51048, respectively, obtained from Shunyou (Shanghai) Biotechnology Co., Ltd.).

The sensitivity of the strains to commonly used antibiotics was determined using the Kirby-Bauer agar disk diffusion method. In a sterile laminar flow hood, individual colonies were inoculated into LB liquid culture and incubated on a shaker at 37 °C until they reached the logarithmic growth phase. The well-cultured bacterial suspension was collected by centrifugation. It was then suspended in sterile PBS, and the bacterial concentration was adjusted to 1 × 10^5^ CFU/mL.

After thoroughly mixing the liquid medium, evenly distribute it onto solid LB agar medium. Place the tested antibiotic paper on the surface of the agar and then incubate it in a biochemical incubator at 37 ℃ for 18–24 h. Subsequently, measure the diameter of the antibiotic inhibition zone using a caliper. as per the drug sensitivity criteria outlined in the M100 guidelines of the Clinical and Laboratory Standards Institute (CLSI), the KP ATCC700603 strain is employed as the quality control strain (Kiffer et al. 2005; Hackel et al. 2019).

### Minimum inhibitory concentration determination

The minimum inhibitory concentrations (MICs) of various antimicrobial drugs were determined using the broth microdilution method recommended by CLSI. The operational procedures and interpretation of results strictly followed the reference standard CLSI M100. The test drugs used in the study included IM, meropenem, chloramphenicol, ciprofloxacin, levofloxacin, ceftazidime, ceftriaxone, cefixime, and aztreonam (catalog numbers: J13049, J13050, J13032, J13015, J13014, J13004, J13002, J13007, J13048; Shanghai, China, Shunyou (Shanghai) Biotechnology Co., Ltd.) The minimum inhibitory concentrations (MICs) of Ertapenem were determined using MIC susceptibility testing strips (92157, Shanghai, China, Shunyou (Shanghai) Biotechnology Co., Ltd.), following the standard procedures for drug susceptibility testing. The quality control strain used is KP ATCC700603 (Hackel et al. 2019; Zhang et al. 2020a; Nordmann et al. 2012).

### Carbonic anhydrase isoenzyme phenotype detection

The experiment utilized the modified carbapenem inactivation method (mCIM) and the EDTA-modified carbapenem inactivation method (eCIM) in accordance with the CLSI M100S standard procedure. Two 5 mL TBS broth tubes (PM0580, Beijing, China, Beijing Coolab Technology Co., Ltd.) were prepared, one for the mCIM experiment and the other for the eCIM experiment. To the eCIM test tube, add 50 μL of a 0.5 M EDTA solution (SL3069, 580, Beijing CooLeBo Technology Co., Ltd., Beijing, China). The overnight culture of the tested KP and the sensitivity paper, containing 10 µg meropenem (Z51050, Shanghai, Shunyou (Shanghai) Biotechnology Co., Ltd.), were inoculated with a 1 µL inoculation loop into the mentioned solutions. The inoculated solutions were then incubated for 4 h ± 15 min. A 1.5 × 10^8^ CFU/mL bacterial suspension was prepared using sterile physiological saline and spread onto an MH agar plate (HBPM6232, Qingdao, Qingdao High-tech Industrial Park Haibo Biotechnology Co., Ltd.). Subsequently, the meropenem paper strips were removed from the mCIM and eCIM test tubes and placed onto the MH above the agar plate. The plate was incubated for 12 h before measuring the diameter of the inhibition zone.

According to the interpretation criteria provided by the Clinical and Laboratory Standards Institute (CLSI), the mCIM test classifies meropenem disks inhibitory zones as follows: if the diameter is 6–15 mm or 16–18 mm with scattered colonies within the zone, it is considered carbapenemase-positive; if the diameter is ≥ 19 mm, it is considered negative; when the inhibitory zone diameter is 16–18 mm or ≥ 19 mm, but with scattered colony growth within the zone, it is considered carbapenemase indeterminate. In the eCIM test, when compared to the results of mCIM, a diameter of ≥ 5 mm in the Meropenem inhibition zone indicates metallo-beta-lactamase positivity, while a diameter of ≤ 4 mm indicates metallo-beta-lactamase negativity. Any isolated needle-point colonies within the inhibition zone of the eCIM could be disregarded (Pierce et al. 2017).

### PCR detection of carbapenemase genotypes

The strain was inoculated onto blood agar medium (024070, Guangzhou, Guangdong Huan Kai Microbiological Technology Co., Ltd.) and incubated overnight at 37 ℃ in a 5% CO2 bacterial incubator. Bacterial DNA templates were extracted using a heat boiling method. Ten microliters of bacterial culture were transferred into 1 mL of sterile deionized water using an inoculation loop. The mixture was then boiled in a dry heat block for 10 min, followed by centrifugation at 12,000 rpm for 10 min. The supernatant containing the DNA was subsequently transferred to a new EP tube for PCR detection. The types of genes and primer sequences tested are shown in Additional file 1: Table S1. The amplification conditions and PCR reaction system were identical to those mentioned previously. After electrophoresis in a 1% agarose gel at 100 V for 30 min, the PCR products were exposed and photographed using a gel imaging system.

### Sample collection and transcriptome sequencing

RNA Extraction and Detection: The KP and CRKP strains were cultured to the logarithmic growth phase and then centrifuged at 12,000 × *g* for 3–5 min at 4 °C to pellet the bacterial cells. Approximately 100 mg of the bacterial pellet was used for total RNA extraction using the RNAiso Plus Total RNA Extraction Kit (9108Q, Takara, Beijing, China), following the instructions. The RNA concentration was determined using the Nanodrop ND-1000 Spectrophotometer (Thermo Fisher), measuring the OD260/280 ratio to ensure a purity range of 1.8–2.0. The RNA concentration was also assessed using the Qubit RNA Assay Kit (Q33221, Thermo Fisher, USA). Total RNA samples meeting the following criteria were used for subsequent experiments: RNA Integrity Number (RIN) ≥ 7.0 and 28S:18S ratio ≥ 1.5.

RNA transcriptome sequencing: The libraries for sequencing were generated and sequenced by CapitalBio Technology (Beijing, China). Each sample uses a total of 5 μg RNA. In brief, the Ribo-Zero Magnetic Kit (MRZG12324, purchased from Epicentre, USA) depletes ribosomal RNA (rRNA) from total RNA. The sequencing libraries were prepared utilizing Illumina's NEB Next Ultra RNA Library Prep Kit (E7760S, procured from NEB, USA). The RNA fragments are then fragmented into approximately 300 base pair (bp) fragments. It is achieved using NEB Next first strand synthesis reaction buffer (5x), reverse transcriptase primers, and random primers for the first strand cDNA synthesis. The second strand cDNA is synthesized in the second strand synthesis reaction buffer containing dUTP Mix (10x). The ends of the cDNA fragments are repaired by adding polyA tails and connecting sequencing adapters. Following the ligation of the Illumina sequencing adapters, the second strand of cDNA was digested with USER Enzyme (M5508, purchased from NEB, USA) to generate a strand-specific library. Amplify the DNA library, followed by purification and enrichment with PCR. Finally, the library was evaluated using the Agilent 2100 machine, and its quantity was determined with the KAPA Library Quantification Kit (kk3605), purchased from Merck in the USA. Finally, we conducted paired-end sequencing using the Illumina NextSeq CN500 sequencer (Ayturk 2019; Simoneau et al. 2021; Zhang et al. 2020b).

### Transcriptome sequencing data analysis

The quality of paired-end reads in raw sequencing data was evaluated using FastQC software v0.11.8. The raw data was preprocessed using Cutadapt software 1.18 to remove Illumina sequencing adapters and poly(A) tail sequences. Remove reads with N content exceeding 5% using a Perl script. Reads with a base quality of 20 or higher, which comprise 70% of the total, were extracted using FASTX Toolkit software version 0.0.13. Use BBMap software to repair paired-end sequences. Lastly, the filtered fragments of high-quality reads were aligned to the reference genome using the hisat2 software version 0.7.12.

Differential expression analysis of mRNA read counts was conducted using the "edgeR" package in R language, applying filtering criteria of |log2FC|> 1 and P value < 0.05. We utilized the KOBAS intelligence tool to conduct the Kyoto Encyclopedia of Genes and Genomes (KEGG) pathway enrichment analysis to investigate the involvement of differentially expressed genes in biological metabolic and signaling pathways. All enrichment analyses were conducted using a significance threshold of FDR < 0.05. Protein–protein interaction (PPI) relationships of key factors were analyzed using the STRING database (https://string-db.org/), with a minimum interaction score set at 0.700. Visualizing protein–protein interaction networks and identifying core genes could be achieved using the Cytoscape 3.5.1 software and its integrated tool, CytoHubba (Wei et al. 2020; Bu et al. 2021).

### CRISPR-Cas9 technology knocks out the blaNDM-1 gene

The aim was to disrupt the blaNDM-1 Gene in CRKP strains using CRISPR/Cas9 technology. sgRNAs targeting the blaNDM-1 Gene were designed using CRISPOR and synthesized by SBS Genetech, Shanghai. The sgRNA sequences were blaNDM-1-sgRNA1: 5ʹ-GATCCAGTTGAGGATCTGGG-3ʹ (PAM: CGG) and blaNDM-1-sgRNA2: 5ʹ-AGCGACTTGGCCTTGCTGTC-3ʹ (PAM: CGG). Recombinant plasmids were created using the PX458 vector, containing an EGFP tag, puromycin resistance, and a BbsI site.

The research focused on editing bacterial genomes using a chemical transformation method with CaCl2 solution. The strains were grown in LB liquid medium to an OD600 of 0.4, cooled to 0 ℃, and centrifuged. Cells were washed with a "Na solution" (5 mM Tris–HCl, 100 mM NaCl, 5 mM MgCl2), then resuspended in a "Ca solution" (5 mM Tris–HCl, 100 mM CaCl2, 5 mM MgCl2) and incubated. After a second centrifugation, the pellet was resuspended in 'Ca solution', combined with 1 ng of plasmid, and incubated sequentially at 4 ℃ and 42 ℃. The mixture was then added to an LB medium and incubated with 4 μg/mL puromycin for selection.

Transfection efficiency in bacterial strains was assessed via flow cytometry and fluorescence microscopy using EGFP fluorescence. Genomic DNA from a blaNDM-1 knockout CRKP strain and a control CRKP strain was extracted for PCR agarose gel electrophoresis, using primers AAGCTGAGCACCGCATTAG and CGCCTCTGTCACATCGAAAT. Gel blocks were sent to Genewiz, Shanghai, for sequencing to confirm the blaNDM-1 Gene knockout (Wang et al. 2022, 2018a; Sapranauskas et al. 2011).

### Detection of bacterial drug resistance

Lines were drawn on an LB agar plate using an inoculating loop, and a small amount of bacteria was inoculated to cultivate a single colony overnight at 37 °C. From the agar plate, 1–2 bacterial colonies were selected using a sterile loop and suspended in 5 mL of physiological saline to achieve an optical density of 0.5 McFarland units. A cotton swab was dipped into the bacterial suspension and evenly spread on an MH agar plate, rotating the plate by 60 degrees after each application, repeated five times.

Carbapenem antibiotics, IM, meropenem, cefotaxime, and amikacin-loaded E-test strips (from Beijing Puyi Technology Co., Ltd.) were handled with tweezers and attached to the bacteria-covered MH agar plate, ensuring the elimination of air bubbles. The petri dish was then placed in a 37 ℃ incubator for 16 h for cultivation, after which the antimicrobial effect was observed (Wu et al. 2019).

### Western blot

RIPA buffer containing 1% PMSF was used for cell lysis and protein extraction, following the instructions in the provided manual. The protein concentration was measured using a BCA assay kit, with adjustments made to reach a 1 μg/μL concentration. Samples of 100 μL were prepared, boiled at 100 °C for 10 min, and then stored at − 80 °C.

An SDS-PAGE gel with a concentration range of 8–12% was utilized for protein separation. In this process, 50 μg of protein per lane was applied, and the gel was run at a constant voltage of 80–120 V for 2 h. Proteins were then transferred to a PVDF membrane using a wet transfer method at 250 mA for 90 min.

At room temperature, the membrane was blocked with 5% skimmed milk in TBST for 1 h, followed by a rinse with TBST. Samples were then incubated with the primary antibody (as listed in Additional file 1: Table S2) overnight at 4 °C, and subsequently washed three times with TBST. The membrane was further incubated with either goat anti-rabbit or anti-mouse IgG HRP-conjugated secondary antibody at a 1:5000 dilution for 1 h at room temperature, followed by three TBST washes. The samples were developed using an ECL solution and imaged with a gel imager. GAPDH was used as an internal control, and the grayscale values of the target and control bands were compared to determine the protein expression levels. Each experiment was repeated thrice (Wang et al. 2018b; Zalucki et al. 2020).

### RT-qPCR for gene expression

Genomic DNA was extracted from bacterial colonies using the PureLink Genomic DNA Mini Kit (K182002, Invitrogen, USA) following the manufacturer's instructions. Real-time fluorescence quantitative PCR was performed using the Rotor-Gene Q Real-Time PCR Detection System (Qiagen, Hilden, Germany), with the rho gene employed as the reference gene. The reaction system was prepared according to the instructions provided with the QuantiTect Probe RT-PCR Kit (204443, Qiagen, Germany), and the reaction program was set accordingly. All RT-qPCR experiments were conducted using triplicate wells and repeated three times. The 2^−ΔΔCt^ method quantifies the fold change in target gene expression in the experimental group compared to the control group. The formula is as follows: ΔΔCT = ΔCt experimental group − ΔCt control group, where ΔCt = Ct target gene − Ct reference gene. Ct represents the number of amplification cycles necessary for the real-time fluorescence intensity to reach the predefined threshold, signifying the start of the exponential growth phase of amplification (Ong et al. 2011; Gomes et al. 2018). The primer design is shown in Additional file 1: Table S3.

### Serum cytotoxicity assay

To dilute KP to 10^6^ CFU/mL, mix it with 0.9% saline. In the experiment, 50 μL of this dilution was added to various solutions: human serum, 25 μM IM, 0.4 μM TH, combinations of IM with TH or cefepime, and different IM concentrations. Cultivation was at 37 ℃, 200 rpm, with hourly bacterial counts for 4 h. Each strain underwent three tests, with colony counts compared at each time point.

For bacterial counting, 500 μL of the suspension was diluted tenfold, then 1 mL was mixed with nutrient agar and incubated at 37 ℃ for 48 h. Colony numbers were recorded to calculate the total bacteria in the original sample (Zhang et al. 2020b; Li et al. 2023b; Choi et al. 2004). Each strain is tested three times.

### Detection of bacterial virulence in a model of wax moth larvae infection

This experiment involved 140 sixth instar wax moth larvae, each 2–3 cm long and weighing 250–300 mg. These larvae were selected for uniform body surface and viability and kept in sawdust at 18 ℃ in darkness before the experiment. The strain cultured in LB broth to the logarithmic phase was adjusted to 1.0 × 10^7^ CFU/mL using phosphate buffer. Using a Hamilton microliter syringe, 10 μL of bacterial solution was injected into each larva's body cavity.

Ten replicates were conducted for each test strain, with 10 larvae in each replicate. The larvae were incubated at 37 ℃ for 4 days in a CO_2_ incubator, and their survival was recorded daily to plot a survival curve.

In Experiment 1, forty larvae were divided into four groups for bacterial strain infections: Normal (control, sterile PBS, n = 10), CRKP (CRKP strain infection, n = 10), blaNDM-1-KO (CRKP with blaNDM-1 gene knockout, n = 10), and KP (KP strain infection, n = 10). Larvae survival was monitored every 12 h for 5 days.

Experiment 2 involved 50 larvae, with the normal control group (n = 10) not infected and treated with sterile PBS. The remaining 40 larvae, infected with CRKP strains, were divided into four groups (n = 10 each): PBS, IM (15 mg/kg), Thaumatin (0.3 mg/kg), and a combination of IM and Thaumatin. Survival was monitored every 12 h for 5 days.

In Experiment 3, from 50 larvae, 10 formed the normal control group, receiving sterile PBS without bacterial infection. The remaining 40, infected with CRKP strains, were divided into four groups (n = 10 each): PBS, IM (16 mg/kg), SU (8 mg/kg), and a combination of IM and SU. Their survival was similarly monitored every 12 h for 5 days (Li et al. 2023b; Choi et al. 2004; Brust et al. 2019).

### Construction of HAP mouse model

A total of 140 male SPF C57BL/6 mice, 6–8 weeks old, were utilized in the study. These mice were purchased from Beijing Vital River Laboratory Animal Technology Co., Ltd. (Beijing, China). The mice weigh between 18 and 25 g and are housed in SPF-grade animal laboratory cages for feeding purposes. The room's lighting control follows a schedule of 12 h of light and 12 h of darkness. Additionally, the humidity is maintained between 60 and 65%, while the temperature ranges between 22 to 25 ℃. Following one week of adaptation feeding, the mice were monitored for their health status prior to commencing the experiment.

The procedure for developing a mouse animal model of infection is as follows: Initially, 2% isoflurane (1 L/min, R510-22-10, RWD Life Science, Shenzhen, China) is added to an O2 mixture to induce profound anesthesia in the mice. The mice were then securely positioned vertically. The larynx was exposed, and a 12-gauge blunt needle was inserted into the trachea. It was followed by the injection of 100 μL/10 g of bacterial suspension with a concentration of 3 × 10^9^ CFU/mL. The vertical position was maintained for 5 min.

Experiment 1: Out of the 140 mice, a random selection of 40 mice was divided into four groups. Each group was infected with different strains of bacteria using the methods described above. The groups included: Normal group (normal control group, intratracheal injection of sterile PBS, n = 10); CRKP group (infection with CRKP strain as empty control, n = 10); blaNDM-1-KO group (infection with CRKP strain knock-out for blaNDM-1, n = 10); KP group (infection with KP strain, n = 10). Subsequently, the survival rate of the mice was observed at a 24-h interval for a total of 7 days.

Experiment 2: Out of the 50 mice, 10 were randomly chosen to form the normal control group (referred to as the Normal group; n = 10). These mice remained uninfected with bacteria and received treatment solely in the form of sterile PBS injection. Forty of the remaining mice were infected with the CRKP strain using the previously mentioned method. Subsequently, they were randomly divided into four groups, each consisting of ten mice. At 24 h post-infection, mice from each group received an intraperitoneal injection of sterile PBS (PBS group, n = 10), 15 mg/kg of IM (IM group, n = 10), 0.3 mg/kg of TH (TH group, n = 10), and a combination of 15 mg/kg of IM and 0.3 mg/kg of TH (IM + TH group, n = 10). Subsequently, the mice's infection level, pathological changes, and survival rate were monitored at 24-h intervals for 7 days.

Experiment 3: Out of the 50 mice, 10 were randomly assigned to the normal control group (Normal group, n = 10). This group did not experience bacterial infection and received only sterile PBS injections for treatment. Forty of the remaining mice were infected with the CRKP strain using the previously mentioned method. Subsequently, they were randomly divided into four groups, each consisting of ten mice. At 24 h post-infection, sterile PBS was injected into the peritoneal cavity of each group of mice in the PBS group (n = 10). The IM group (n = 10) received intramuscular injections of IM at a dose of 16 mg/kg. The SU group (n = 10) received injections of cefoperazone at a dose of 8 mg/kg. The IM + SU group (n = 10) received a combination of IM at 16 mg/kg and cefoperazone at 8 mg/kg. Subsequently, infection level and survival rate of mice were monitored at 24-h intervals for 7 consecutive days.

A hematological analysis was carried out seven days following the induction of bacterial infection in mice. An equivalent number of mice were arbitrarily chosen from each experimental group for the purposes of enucleation and blood sample collection, predicated on the final survival rate. A volume of 200 μL of whole blood containing EDTA-K2 anticoagulant (G0280, Beijing Solabao Technology Co., Ltd.) was extracted, and a comprehensive blood count analysis was subsequently performed using the BM830 automated hematology analyzer (Beijing Baolingman Suntech Co., Ltd.) to determine the levels of white blood cells and neutrophils.

Colony counts within the pulmonary tissue homogenate were conducted as follows: Following euthanasia of the mice, lung tissues were isolated. Tissue (1 g) was transferred into a tissue homogenization tube, and 1 mL of pre-cooled saline solution was added. Subsequently, a lung tissue homogenate was prepared at a temperature of 4 ℃. The tissue homogenate was then diluted to a 108-fold dilution using a gradient of physiological saline solution at a concentration 10^8^-fold higher. A volume of 0.1 mL from each dilution of the homogenized slurry was evenly spread on blood agar plates, with two plates used for each concentration. The plates were then placed in a 37 ℃ incubator for 24 h to facilitate the enumeration of bacterial colonies (Ma et al. 2019; Li et al. 2023b).

### ELISA

After the collection of serum samples from various groups of mice, the ELISA assay kit was utilized to assess the levels of C-reactive Protein (CRP), procalcitonin (PCT), interleukin (IL)-1β, and IL-6 expression in the mouse serum, following the instructions provided by the manufacturer of the reagent kits (E-EL-M0053c, E-EL-M2419c, E-EL-M0037c, and E-EL-M0044c). These reagent kits were sourced from Elabscience, a company headquartered in Wuhan, China. Optical density (OD) was measured at 450 nm (Kumar et al. 2020).

### H&E staining

A portion of lung tissue was fixed in 4% paraformaldehyde, followed by dehydration and transparency. The tissue was then embedded in paraffin. Slices with a thickness of 5 μm were made using a microtome and subjected to baking and dewaxing until the slices were immersed in water. Subsequently, following the instructions provided, lung tissue pathology was observed using a hematoxylin–eosin staining kit (C0105S, Beyotime, Shanghai, China). Finally, morphological changes in mouse lung tissue were examined using an optical microscope (Ma et al. 2019).

### Statistical analysis

The data were collected from at least three independent experiments and presented as mean ± SD. To compare two sets of data, employ the independent samples t-test, while to compare three or more sets of data, utilize the one-way analysis of variance (ANOVA). If the analysis of variance yields differences, it is necessary to conduct additional post-hoc tests, such as Tukey's HSD, to compare the differences among the groups. The Mann–Whitney U test was used for data that is not normally distributed or heteroscedastic. Statistical analyses were conducted using GraphPad Prism 9.5.0 (GraphPad Software, Inc.) and R 4.2.1 (R Foundation for Statistical Computing). Utilize Pearson correlation analysis to examine the correlation among indicators. The significance level for all tests is set at 0.05. A two-sided p-value lower than 0.05 is considered to indicate a statistical difference.

## Results

### Isolation and characterization of carbapenem-sensitive and -resistant KP strains from HAP patients

**Fig. 1:**
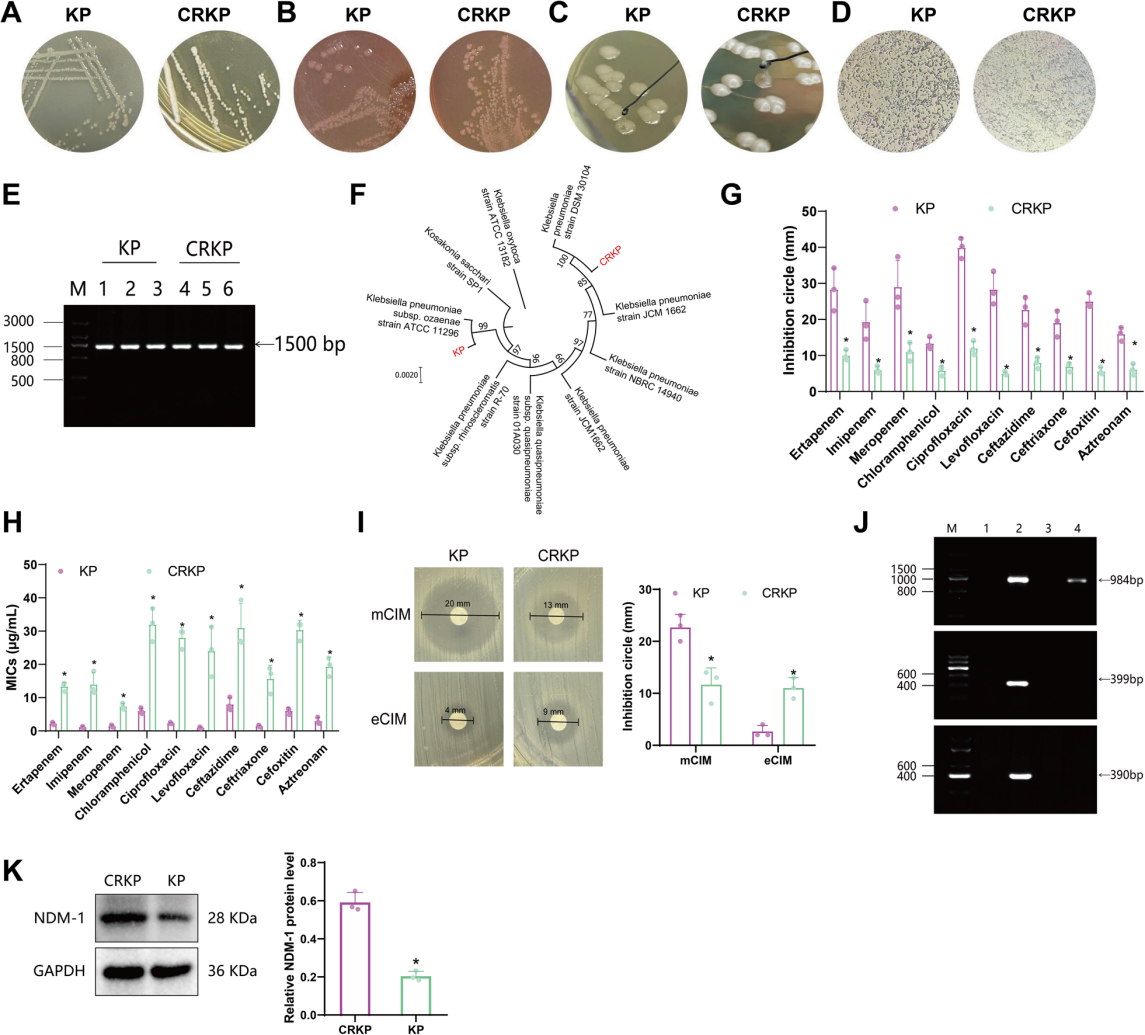
Isolation and identification of KP and CRKP. **A** Colony morphology of KP and CRKP strains on LB agar plates. **B** Colony morphology of KP and CRKP strains on MacConkey agar. **C** Detection of high viscosity phenotype in KP and CRKP strains. **D** Gram staining results of KP and CRKP strains (1000 × magnification). **E** PCR gel electrophoresis detection of 16S rRNA gene amplification products in KP and CRKP strains (Lane M: DNA molecular weight marker; Lanes 1, 2, 3: amplification products of KP 16S rRNA gene; Lanes 4, 5, 6: amplification products of CRKP 16S rRNA gene). **F** Phylogenetic tree analysis of 16S rRNA gene in KP and CRKP strains. **G** Kirby-Bauer paper disk diffusion method for detecting the inhibition zones of KP and CRKP strains against 10 antibiotics (ertapenem, IM, meropenem, chloramphenicol, ciprofloxacin, levofloxacin, ceftazidime, ceftriaxone, cefotaxime, and amikacin). **H** Broth microdilution method for detecting the MICs of KP and CRKP strains against 10 antibiotics (ertapenem, IM, meropenem, chloramphenicol, ciprofloxacin, levofloxacin, ceftazidime, ceftriaxone, cefotaxime, and amikacin). **I** Modified carbapenem inactivation method (mCIM) for detecting carbapenemase phenotype in KP and CRKP strains, and ethylenediaminetetraacetic acid-carbapenem inactivation method (eCIM) for detecting metallo-β-lactamase phenotype in KP and CRKP strains. **J** PCR gel electrophoresis detection of carbapenemase genes in KP and CRKP strains (Lane M: DNA molecular weight marker; Lane 1: negative control; Lane 2: positive control; Lane 3: KP strain; Lane 4: CRKP strain). **K** Western blot experiment to detect the expression levels of NDM1 protein in KP and CRKP strains (upper figure), as well as the statistical results (lower figure). Quantitative data in the figure are represented as mean ± SD, and each experiment group was repeated three times. *P < 0.05 compared to the KP group

KP is one of the major pathogens associated with HAP (Li et al. 2023a). Therefore, we selected a patient undergoing treatment with carbapenem antibiotics and another patient who has not received carbapenem therapy based on the clinical and microbiological diagnostic criteria for HAP. We isolated KP and carbapenem-resistant KP (CRKP) strains from their blood samples. After incubating the isolated strains at 37 °C for 24 h, we observed uniform, moist, smooth-edged milky white mucoid colonies of KP and CRKP on LB agar plates (Fig. 1A). On MacConkey agar medium, the colonies appeared pink, moist, and round (Fig. 1B).

We conducted high-viscosity phenotype testing on strains of KP and CRKP. The results demonstrated that when the colonies were stretched on agar plates with an inoculation loop, the length of the mucoid material exceeded 5 mm, indicating a high viscosity (Fig. 1C). These findings align with the colony morphology characteristics typically observed in KP. Gram staining microscopy revealed that both KP and CRKP colonies exhibited red oval shapes, indicating the presence of Gram-negative rods (Fig. 1D).

Furthermore, the biochemical test results indicated that both KP and CRKP strains exhibited positive reactions for urea, 7-leucine, lactose, glucose, sucrose, maltose, cysteine desulfhydrase, and V-P test. However, they showed negative reactions for the ornithine decarboxylase and indole test, which is consistent with the biochemical reaction characteristics of KP (Table 1).

**Table 1:** Biochemical characterization of isolates

Measurement items	Result					
	KP			CRKP		
Urea	+	+	+	+	+	+
Hesperidin	+	+	+	+	+	+
Lactose	+	+	+	+	+	+
Glucose	+	+	+	+	+	+
Fructose	+	+	+	+	+	+
Maltose	+	+	+	+	+	+
Hydrogen sulfide	+	+	+	+	+	+
Lysine decarboxylase	+	+	+	+	+	+
Ornithine decarboxylase	−	−	−	−	−	−
V-P test	+	+	+	+	+	+
Ehrlich indol test	−	−	−	−	−	−

“ + ” indicates a positive test result; “−” indicates a negative test result, and each set of experiments was repeated 3 times

Subsequently, bacterial genomes were extracted, followed by PCR amplification of the 16S rRNA gene. Amplification products were then detected using agarose gel electrophoresis. The results demonstrated that the PCR amplification products obtained from the KP and CRKP strains displayed distinct bands at approximately 1500 bp (Fig. 1E), which aligned with the anticipated outcomes.

The amplified products were sequenced and compared to the NCBI database for analysis. The sequences of the two strains showed similarity to KP. The genetic evolutionary tree was constructed using MEGA11 software (Fig. 1F). The results revealed that the isolated KP strains had a 99% homology with KP subsp. ATCC11296 and a 100% homology with subsp. DSM30104. The results suggest successful isolation of KP from the blood of patients with HAP.

In addition, we utilized the K-B paper disk diffusion method, as outlined in the Clinical and Laboratory Standards Institute (CLSI) M100 document, to assess the susceptibility of KP and CRKP strains to ten commonly prescribed antibiotics (ertapenem, IM, meropenem, chloramphenicol, ciprofloxacin, levofloxacin, cefotaxime, ceftriaxone, cefepime, and amikacin) (Fig. 1G). The results indicated that CRKP strains exhibited smaller inhibition zones than KP strains for all ten antibiotics. Based on the MIC thresholds and breakpoints provided in the CLSI M100 document, strains of KP are found to be susceptible to all 10 antimicrobial drugs, while CRKP exhibits resistance to all 10 antimicrobial drugs (Table 2).

**Table 2:** Susceptibility of KP and CRKP strains to 10 antimicrobial drugs

Antibiotics	Drug content of paper /μg	Inhibitory ring diameter (mm)				
		R (mm)	I (mm)	S (mm)	KP (mm)	CRKP (mm)
Ertapenem	10	≤ 18	19–21	≥ 22	24.1 ± 1.5	10.0 ± 1.4*
Imipenem	10	≤ 19	20–22	≥ 23	26.2 ± 1.9	5.9 ± 1.1*
Meropenem	10	≤ 19	20–22	≥ 23	26.1 ± 2.4	11.5 ± 2.2*
Chloramphenicol	30	≤ 12	13–17	≥ 18	20.0 ± 1.5	5.9 ± 0.9*
Ciprofloxacin	5	≤ 21	22–25	≥ 26	29.9 ± 2.9	12.6 ± 1.9*
Levofloxacin	5	≤ 16	17–20	≥ 21	24.9 ± 2.0	5.0 ± 0.6*
Ceftazidime	30	≤ 17	18–20	≥ 21	26.0 ± 2.6	8.8 ± 1.3*
Ceftriaxone	30	≤ 19	20–22	≥ 23	28.1 ± 2.4	6.8 ± 1.1*
Cefoxitin	30	≤ 14	15–17	≥ 18	25.2 ± 2.0	5.8 ± 0.8*
Aztreonam	30	≤ 17	18–20	≥ 21	26.0 ± 2.2	9.1 ± 1.5*

*R* Resistance, *I* Intermediary, *S* Sensitive. The measured data in the figure is presented as Mean ± SD, with 3 times in each group. *Represents a significant difference compared to the PBS group, *P < 0.05

The broth microdilution method determined the MIC values of 10 antimicrobial drugs against KP and CRKP strains. The results indicated that the MIC values of CRKP strains were higher than those of KP strains for all 10 drugs (Fig. 1H). Furthermore, based on the CLSI M100 susceptibility criteria, it was confirmed that KP strains were susceptible to all 10 antimicrobial drugs, while CRKP strains were resistant to all 10 drugs (Table 3).

**Table 3:** MICs of KP and CRKP strains against 10 antimicrobial drugs

Antibiotics	MICs (μg/mL)				
	R (μg/mL)	I (μg/mL)	S (μg/mL)	KP (μg/mL)	CRKP (μg/mL)
Ertapenem	≥ 2	1	≤ 0.5	0.2 ± 0.1	18.5 ± 4.1*
Imipenem	≥ 4	2	≤ 1	0.8 ± 0.4	16.1 ± 3.3*
Meropenem	≥ 4	2	≤ 1	1.2 ± 0.7	20.0 ± 3.9*
Chloramphenicol	≥ 32	16	≤ 8	5.2 ± 0.9	31.8 ± 5.5*
Ciprofloxacin	≥ 1	0.5	≤ 0.25	0.2 ± 0.1	28.2 ± 3.1*
Levofloxacin	≥ 2	1	≤ 0.5	0.5 ± 0.2	30.1 ± 5.0*
Ceftazidime	≥ 16	8	≤ 14	8 ± 1.9	27.8 ± 2.3*
Ceftriaxone	≥ 4	2	≤ 1	1 ± 0.8	19.6 ± 1.9*
Cefoxitin	≥ 32	16	≤ 8	5 ± 0.8	32.2 ± 4.7*
Aztreonam	≥ 16	8	≤ 4	3 ± 0.8	26.0 ± 4.6*

*R* Resistance, *I* Intermediary, *S* Sensitive. The measured data in the figure is presented as Mean ± SD, with 3 times in each group. *Represents a significant difference compared to the PBS group, *P < 0.05

The carbapenem phenotype of KP and CRKP strains was detected using the modified Carbapenem Inactivation Method (mCIM) assay. The results revealed a negative carbapenem phenotype for KP strains, whereas CRKP strains exhibited a positive carbapenem phenotype. Additionally, we employed the eCIM assay to determine the metalloenzyme phenotypes of KP and CRKP strains. Our findings revealed that KP strains exhibited a negative metallo-enzyme phenotype, whereas CRKP strains displayed a positive metallo-enzyme phenotype (Fig. 1I).

Hence, metalloenzyme-related genes in KP and CRKP strains were identified using PCR electrophoresis. The findings indicated the presence of the NDM gene in CRKP strains, while the VIM and IMP genes were not detected. KP strains did not exhibit any metalloenzyme-related genes (Fig. 1J). Furthermore, the Western blot analysis revealed a significant increase in the expression of the NDM-1 protein in CRKP strains compared to KP strains (Fig. 1K).

In summary, we successfully isolated carbapenem-sensitive and -resistant strains of KP from blood samples obtained from patients diagnosed with HAP. Furthermore, we discovered that CRKP was positive for carbapenemase and expressed the NDM gene.

### Transcriptomic analysis reveals central role of blaNDM-1 gene in carbapenem resistance among CRKP strains from HAP patients

According to reports in the literature, β-lactam antibiotics are currently the most widely utilized antimicrobial drugs in clinical practice, constituting more than 50% of global antibiotic consumption. Bacteria harboring the NDM-1 metallo-beta-lactamase enzyme can hydrolyze nearly all commonly used beta-lactam antibiotics, such as penicillin, cephalosporins, and carbapenems. This resistance mechanism is considered the most crucial among Gram-negative bacteria (Bahr et al. 2021).

**Fig. 2:**
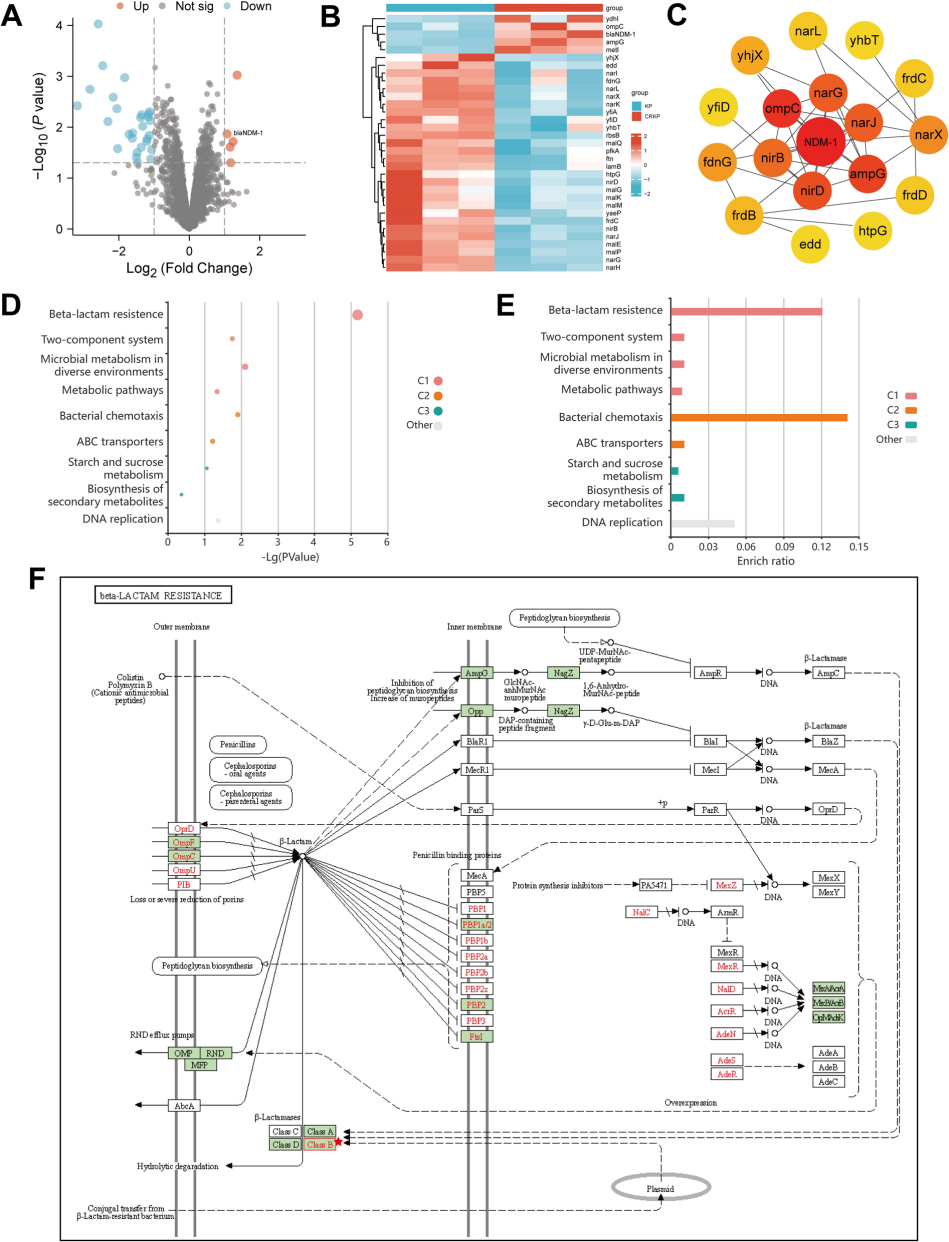
Transcriptional sequencing analysis of potential drug resistance mechanisms in CRKP. **A** Volcano plots of gene expression in three KP strains and three CRKP strains. Orange dots represent upregulated genes, blue dots represent downregulated genes, and black dots represent non-differential genes. **B** Heatmap of differentially expressed genes between KP and CRKP strains. Orange represents upregulated genes, and blue represents downregulated genes. **C** The protein–protein interaction network of NDM-1 protein (Combined score = 0.7), with color ranging from yellow to red, indicates the Degree values of genes from small to large. **D** Bubble plot of KEGG pathway enrichment analysis of differentially expressed genes between KP and CRKP strains. **E** Bar chart of KEGG pathway enrichment analysis results related to beta-lactam resistance and associated pathways in differentially expressed genes between KP and CRKP strains. **F** The content related to Beta-lactam resistance and its associated pathways in the KEGG pathway enrichment analysis results (from the KEGG database at https://www.kegg.jp/kegg/pathway.html)

To better understand the role of NDM-1 in drug resistance among CRKP strains, we performed high-throughput transcriptome sequencing analysis (RNA-seq) on isolated KP and CRKP strains. Three replicates were conducted for each strain to investigate differential gene expression in KP and CRKP strains. Following quality control and filtering of the raw data, differential analysis was conducted using the criteria of |log2FC|> 1 and P.value < 0.05. 33 genes were identified as differentially expressed, comprising 28 downregulated and 5 upregulated genes. Notably, the expression of the gene blaNDM-1, which encodes NDM-1, was upregulated in the CRKP strain (Fig. 2A, B).

We analyzed the protein–protein interaction network of key factors using the STRING database. The interactions were visualized using Cytoscape 3.5.1 software. Additionally, we employed the built-in tool CytoHubba to identify the core proteins. The results demonstrate the roles played by proteins such as NDM-1, ompC, ampG, narG, narJ, and nirD in this network. The NDM-1 protein holds a central position, as indicated by the highest combined score (Fig. 2C).

Based on the analysis of differential genes, we conducted a KEGG pathway enrichment analysis on the obtained differentially expressed genes. The results revealed that these genes were mainly associated with signal transduction pathways, such as Beta-lactam resistance, Two-component system, Microbial metabolism in diverse environments, Metabolic pathways, Bacterial chemotaxis, and DNA replication (Fig. 2D). Notably, the pathways of Bacterial chemotaxis and Beta-lactam resistance exhibited a high enrichment of differential genes (Fig. 2E). Figure 2F illustrates Beta-lactam resistance and its related signal pathways, and previous research has indicated that the Beta-lactam resistance signaling pathway plays a crucial role in conferring carbapenem resistance in KP strains (Zapun et al. 2008). These findings demonstrate that the activation of the Beta-lactam resistance signaling pathway is the primary cause of acquired resistance in CRKP strains. Furthermore, the key signaling molecule in this pathway is β-Lactamases, with Class B proteins composed of the precursor blaNDM-1 encoded by the blaNDM-1_1, blaNDM-1_2, and blaNDM-1_3 genes. Therefore, these results further support the significant role of the blaNDM-1 gene in facilitating acquired resistance in CRKP strains.

The results revealed an upregulation of blaNDM-1 gene expression levels in the resistant strain CRKP compared to the susceptible strains. This difference was found to be statistically significant. This finding suggests that the blaNDM-1 gene might have an essential regulatory function in CRKP strains, closely related to the emergence of carbapenem resistance.

### Targeted knockout of blaNDM-1 gene in CRKP strains diminishes carbapenem resistance and reduces bacterial virulence in vitro and vivo

**Fig. 3:**
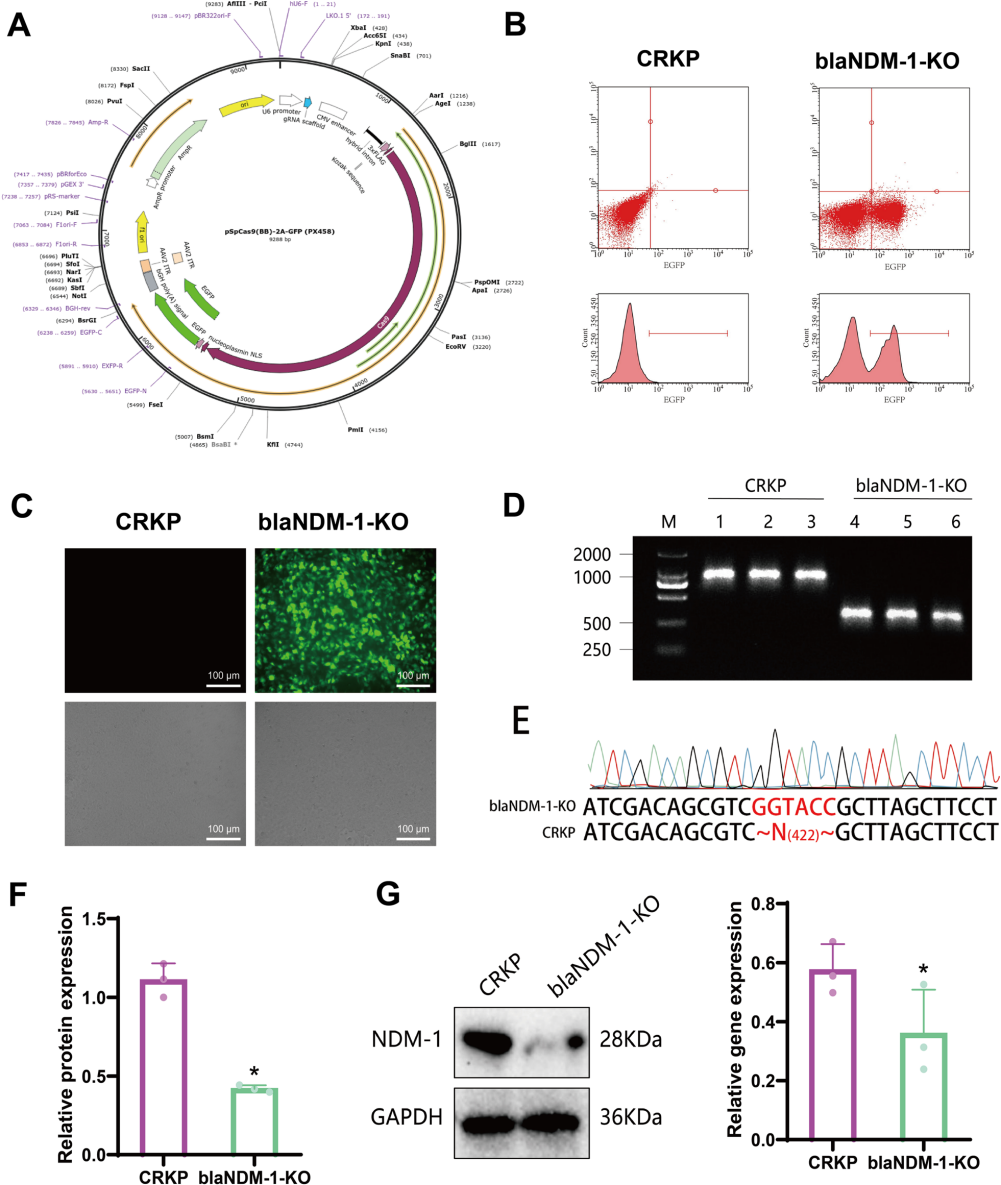
CRISPR-Cas9 Technique Knocking Out the blaNDM-1 Gene in CRKP Strains. **A** Structure of the plasmid vector PX458 (pSpCas9(BB)-2A-GFP). **B** Flow cytometry to detect transfection efficiency of the plasmid vector. **C** Fluorescence microscopy to detect transfection efficiency of the plasmid vector (upper: fluorescence microscope image; lower: light microscope image, scale bar: 100 μm). **D** PCR agarose gel electrophoresis to detect the silencing effect of the blaNDM-1 Gene (M: DNA molecular weight marker; 1, 2, 3: gene amplification products of CRKP strains; 4, 5, 6: gene amplification products of blaNDM-1-KO strains, N represents the number of deleted amino acids). **E** PCR block sequencing results. **F** RT-qPCR to detect the expression of the blaNDM-1 gene. **G** Western blot to detect the expression of NDM-1 protein. Quantitative data in the figure are represented as mean ± SD, with each experiment repeated 3 times. *P < 0.05 indicates significance compared to the CRKP group

To further validate the impact of the blaNDM-1 gene on drug resistance in CRKP strains, we employed CRISPR-Cas9 technology to knock out the blaNDM-1 gene in CRKP strains. Plasmid transfection efficiency was evaluated using flow cytometry and fluorescence microscopy (Fig. 3A). Results demonstrated a significant increase in EGFP green fluorescence in the blaNDM-1-KO CRKP strain compared to the empty vector control. This finding suggests successful transfection of the plasmid vector into the CRKP strain (Fig. 3B, C).

Furthermore, PCR agarose gel electrophoresis detection and sequencing results indicated that the blaNDM-1 Gene was absent in the blaNDM-1-KO group strains, in contrast to the CRKP group (Fig. 3D, E). This finding suggests successful knockout of the blaNDM-1 gene in the CRKP strains. The RT-qPCR detection results revealed a down-regulation of the blaNDM-1 gene expression in the blaNDM-1-KO group bacteria compared to the CRKP group (Fig. 3F).

Western blot analysis revealed a decrease in NDM-1 protein expression in bacterial strains of the blaNDM-1-KO group compared to the CRKP group (Fig. 3G). This result further confirms the successful knockout of the blaNDM-1 Gene in CRKP strains.

**Fig. 4:**
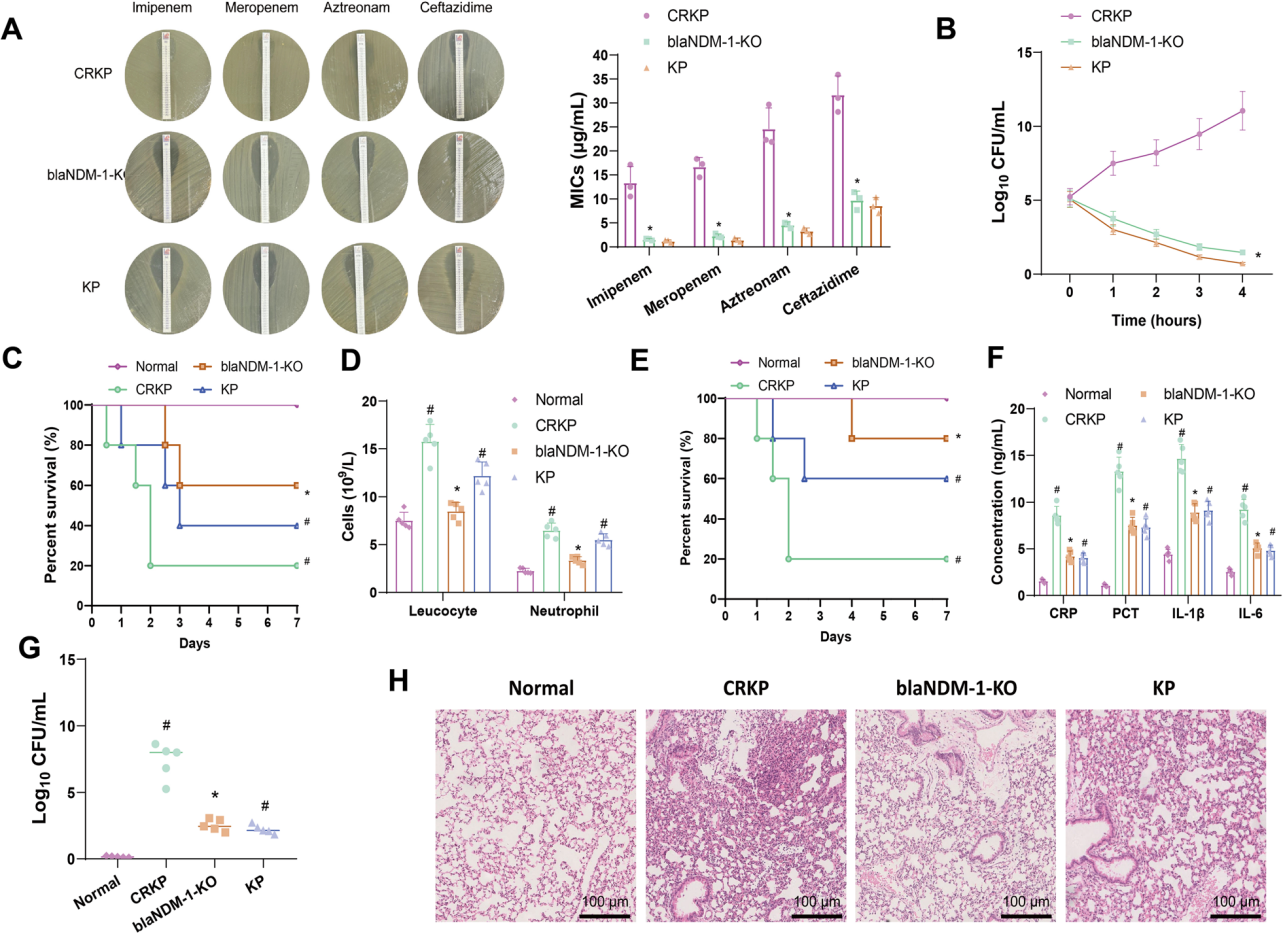
Effect of knocking out the blaNDM-1 gene on the virulence of CRKP strains. **A** Different strains' Minimum inhibitory concentrations (MICs) against IM, meropenem, ceftazidime, and amikacin determined by E-test. **B** Serum bactericidal experiment to assess the effect of silencing the blaNDM-1 gene on the cytotoxicity of CRKP strains. **C** Impact of different strains on the survival rate of Galleria mellonella larvae (n = 10). **D** Comparison of the number of white blood cells and neutrophils in mouse blood 7 days after infection with different strains (n = 5). **E** Evaluation of the impact of different strains on mouse survival rate (n = 10). **F** ELISA to detect the expression of inflammatory factors CRP, PCT, IL-1β, and IL-6 in mouse plasma 7 days after infection with different strains (n = 5). **G** Enumeration of bacteria in mouse lung tissue 7 days after infection with different strains (n = 5). **H** H&E Staining of mouse lung tissue 7 days after infection with different strains (scale bar: 100 μm, n = 5). Quantitative data in the figure are represented as mean ± SD, with each experiment repeated 3 times. *P < 0.05 indicates significance compared to the CRKP group; # indicates significance compared to the Normal group, #*P* < 0.05

Subsequently, we conducted MIC tests on various strains of CRKP using E-test antibiotic sensitivity strips to examine their susceptibility to IM, meropenem, aztreonam, and ceftazidime. The results revealed that the blaNDM-1-KO CRKP strains showed considerably lower MICs for all four antibiotics, similar to the sensitive KP strain (Fig. 4A).

Results from the serum-killing experiment demonstrated that the blaNDM-1-KO CRKP strain exhibited lower viability than the CRKP group. The findings of this exploration suggest that the elimination of the blaNDM-1 Gene in CRKP strains leads to a reduction in the virulence of CRKP strains in vitro (Fig. 4B).

To further confirm the influence of blaNDM-1 gene knockout on the in vivo virulence of CRKP strains, we injected the strains into the larvae of Galleria mellonella and monitored their survival. The results revealed that the larvae from the CRKP and KP groups began dying 12 h post-bacterial infection, whereas those from the blaNDM-1-KO group started dying after 24 h. Furthermore, the survival rates of larvae in the CRKP and KP groups were lower compared to the normal control group. However, the survival rate of larvae in the blaNDM-1-KO group was increased compared to the CRKP group and was nearly identical to that of the carbapenem-sensitive strain KP group (Fig. 4C).

A mouse model was also established by infecting it with various strains through nasal instillation. After 7 days of bacterial infection, the mice were euthanized, and blood samples were collected from their eyeballs for blood routine analysis. The study findings revealed that the infected mice exhibited higher white blood cell and neutrophil counts in their blood compared to the normal control group (Normal group). These differences were statistically significant (P < 0.05) and surpassed the range of normal theoretical values (Fig. 4D). These results indicate that mice infected with bacteria experience inflammation, providing preliminary evidence for constructing a successful infection model. The blaNDM-1-KO group of mice exhibited a decrease in white blood cell and neutrophil count compared to the CRKP group and a reduction in the severity of inflammation (Fig. 4D).

Survival analysis of mice revealed that both the CRKP and KP groups experienced mortality one day after infection, while mice in the blaNDM-1-KO group began to die after two days. Compared to the normal control group, the survival rate of mice in the KP and CRKP groups decreased. Additionally, compared with the CRKP group, the survival rate of mice in the blaNDM-1-KO group increased (Fig. 4E). The findings of the study suggest that the suppression of the blaNDM-1 Gene in CRKP strains could result in a reduction of their virulence.

Recent studies have indicated that the primary mechanism underlying severe pneumonia is the overexpression of cytokines in the body and their associated interactions. The body produces chemotactic factors and cytokines in response to bacterial infection, which are important in the adjunctive diagnosis of bacterial infections (Sandquist and Wong 2014).

The experimental results indicate that, compared to the normal control group, the levels of CRP, PCT, and inflammatory factors (IL-1β and IL-6) in the plasma of mice infected with KP and CRKP were elevated, showing a close correlation with prognosis. In comparison to the CRKP group, the expression of PCT, CRP, IL-1β, and IL-6 in the plasma of mice in the blaNDM-1-KO group was significantly reduced, indicating a weakened inflammatory response in the mice (Fig. 4F).

Bacterial culture experiments were conducted on lung tissues obtained from two groups of mice: the infection group and the control group. The results showed that the lungs of mice in the infection group exhibited white, consistently sized, smooth, and moist colonies of KP, which were well-defined. In contrast, the control group yielded no colonies of KP. Bacterial colony counting was conducted by homogenizing lung tissue to ascertain the bacterial load per Gram of lung tissue. The colony count results indicate an increase in bacterial count in the lung tissue of mice from the KP and CRKP groups, as compared to the normal group. The bacterial load in the lung tissues of blaNDM-1-KO mice was lower compared to the CRKP group (Fig. 4G).

Additional pathology results from H&E Staining of lung tissue revealed that compared to the normal group, the lung tissue of mice from the KP and CRKP groups exhibited infiltration of inflammatory cells. The alveolar walls were also found to be damaged, leading to the shedding of epithelial cells. Moreover, diffuse red blood cells were visible in the alveolar walls and cavities. No abnormalities were observed in the normal control group. In comparison to the CRKP group, the blaNDM-1-KO group exhibited a reduction in the degree of lung tissue abnormality (Fig. 4H).

The results above indicate successful knockout of the blaNDM-1 gene in the CRKP strain using the CRISPR-Cas9 interference technique. After disrupting the blaNDM-1 gene, the drug resistance of CRKP strains towards carbapenem antibiotics decreased significantly, exhibiting a sensitivity comparable to that of susceptible strains.

#### The combination of IM and TH greatly enhances the antibacterial effect

**Fig. 5:**
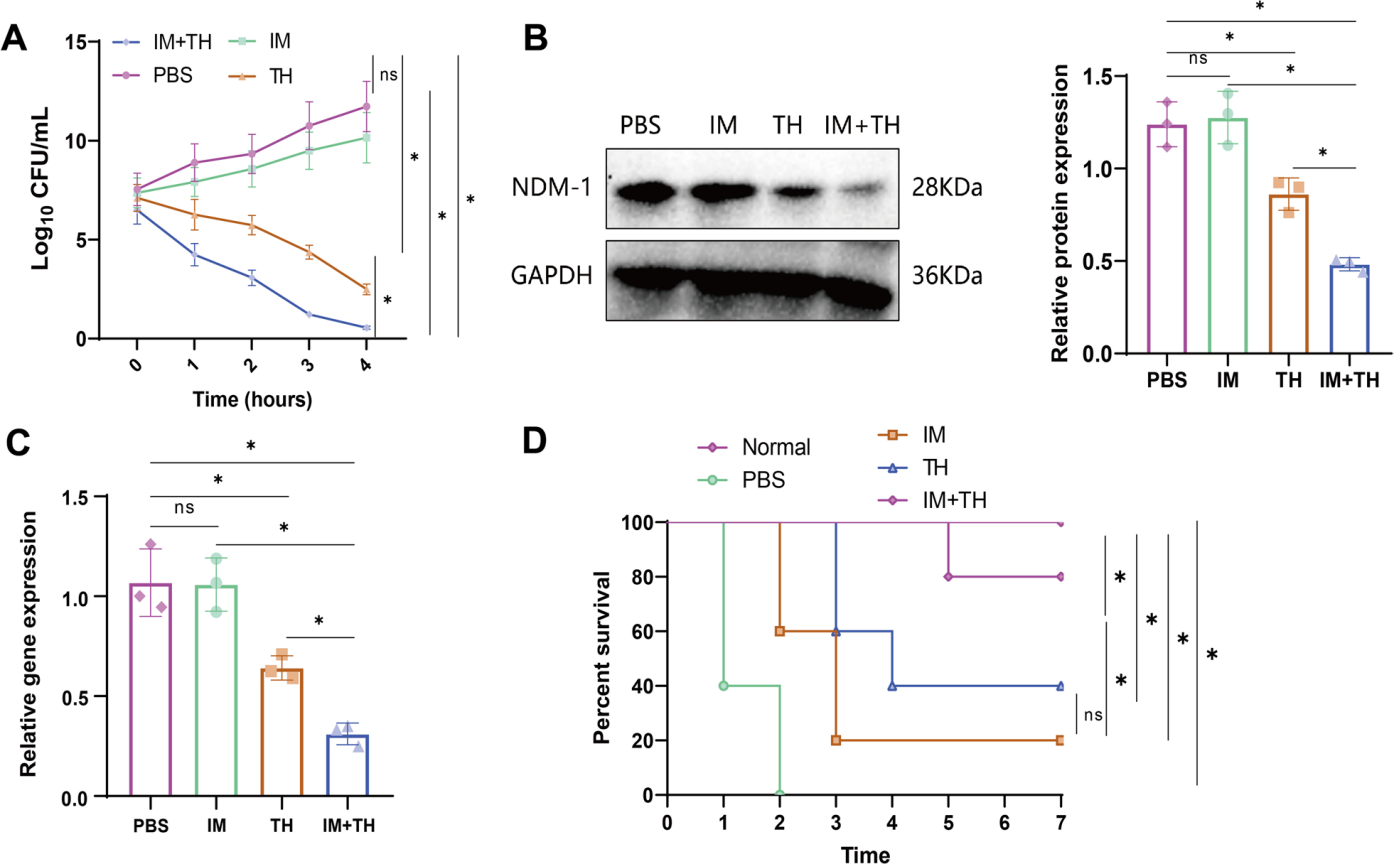
In vitro Inhibition of CRKP Strain Virulence by Combined Treatment with IM and TH. **A** Impact of 25 μM IM, 0.4 μM TH, and the combination of 25 μM IM and 0.4 μM TH on the virulence of CRKP strains. **B** Western blot to detect changes in NDM-1 protein expression in CRKP strains after different drug treatments. **C** RT-qPCR to detect changes in blaNDM-1 gene expression in CRKP strains after different drug treatments. **D** Impact of IM, TH, and IM + TH on the survival rate of Galleria mellonella larvae infected with CRKP strains (n = 10). Quantitative data in the figure are represented as mean ± SD, with each experiment repeated 3 times. *P < 0.05 indicates significance between the two groups; ns indicates no difference between the two groups

Initially, we examined the impact of administering IM and TH, individually and in combination, on the proliferation of CRKP strains. The results indicated that compared to the IM group and TH group, the combined IM and TH treatment group (IM + TH) improved its ability to inhibit the proliferation of CRKP strains (Fig. 5A).

The results of Western blot analysis indicated that the expression of NDM-1 protein in CRKP strains of the IM group remained relatively stable, with little change, when compared to the PBS group. In contrast, TH treatment suppressed the expression of NDM-1 protein. Compared to the IM and TH groups, the combined treatment of IM and TH resulted in an inhibition of NDM-1 protein expression (Fig. 5B). Further validation using RT-qPCR confirmed that the combined treatment with IM and TH suppressed the expression of the blaNDM-1 Gene in CRKP strains (Fig. 5C).

Secondly, we employed intramuscular (IM) and topical hormone (TH) administration to treat wax moth larvae infected with CRKP strains. The results indicate that the waxworm larvae in the PBS group experienced mortality following infection with the CRKP strain, unlike the normal control group. Following treatment with IM and TH, there has been a decrease in the mortality rate of the larvae. Compared to administering IM and TH separately, the combined administration increases the survival rate of wax moth larvae infected with CRKP bacteria (Fig. 5D).

**Fig. 6:**
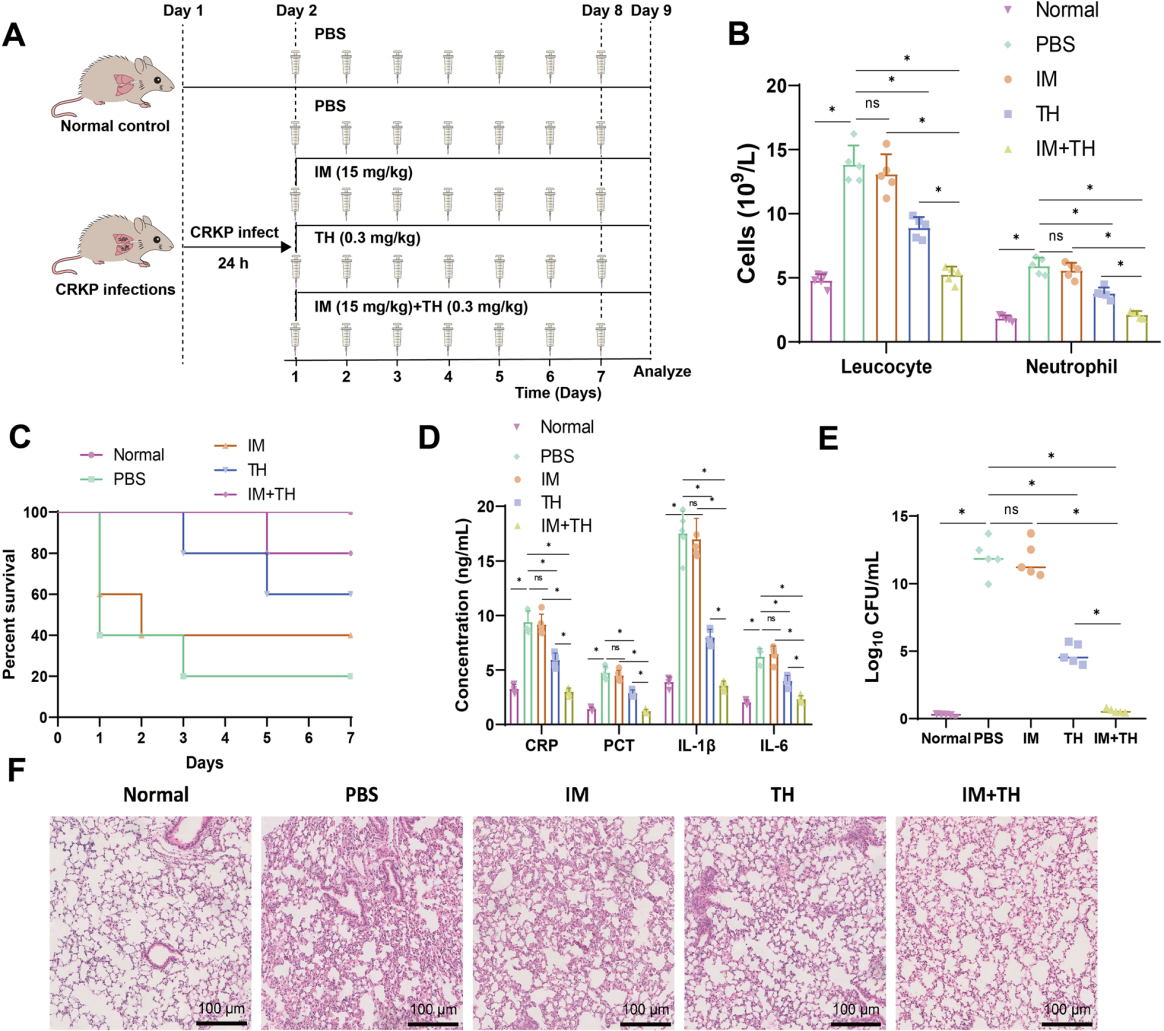
In vivo inhibition of CRKP strain virulence by combined treatment with IM and TH. **A** Illustration of mouse drug treatment. **B** Number of white blood cells and neutrophils in mouse blood after 7 days of IM, TH, and IM + TH treatment in CRKP-infected mice (n = 5). **C** Impact of IM, TH, and IM + TH on the survival rate of CRKP-infected mice (n = 10). **D** ELISA to detect the expression of inflammatory factors CRP, PCT, IL-1β, and IL-6 in mouse plasma after 7 days of IM, TH, and IM + TH treatment in CRKP-infected mice (n = 5). **E** Bacterial count in mouse lung tissue after 7 days of IM, TH, and IM + TH treatment in CRKP-infected mice (n = 5). **F** H&E Staining of mouse lung tissue after 7 days of IM, TH, and IM + TH treatment in CRKP-infected mice (scale bar: 200 μm, n = 5). Quantitative data in the figure are represented as mean ± SD, with each experiment repeated 3 times. *P < 0.05 indicates significance between the two groups; ns indicates no difference between the two groups

Furthermore, we investigated the therapeutic effects of monotherapy with IM and TH and combination therapy on mice infected with CRKP strains (Fig. 6A). The blood routine analysis study revealed higher white blood cell and neutrophil counts in the infected group of mice compared to those in the normal control group. These findings confirm the successful establishment of a mouse model for CRKP infection.

The number of white blood cells and neutrophils in the blood of mice in the IM + TH group decreased compared to the IM group and the TH group. This reduction suggests a decrease in inflammation levels (Fig. 6B). The monitoring results revealed that the mice in the infection group died while those in the treatment group displayed improved survival. Compared to IM and TH alone, the combined treatment effectively enhanced the survival rate of mice infected with CRKP strains (Fig. 6C).

The detection of inflammatory factors PCT, CRP, IL-1β, and IL-6 in mouse plasma revealed an increase in their expression in mice infected with CRKP strain compared to the normal control group when treated with PBS. The mice in the treatment group exhibited reduced expression of inflammatory factors. The combined use of IM and TH exhibits a notably more potent inhibitory effect on the expression of inflammatory factors than their administration. Furthermore, this illustrates that the concurrent administration of IM and TH suppresses the inflammation induced by CRKP infection (Fig. 6D).

After homogenizing the mouse lung tissues, colony counts were performed, which indicated an increase in bacterial quantity in the lung tissues of the mice from the PBS group compared to the normal control group. Following a successful treatment, the bacterial count in the lung tissue of the mice was decreased. The combined use of IM and TH drugs exhibits an enhanced ability to inhibit the proliferation of CRKP strains in the body, surpassing the individual use of either IM or TH drugs alone (Fig. 6E).

The results of H&E Staining on lung tissue reveal that the mouse lung tissue in the PBS group exhibits an increase in inflammatory cell infiltrations, destruction of the alveolar wall structure, and scattered red blood cells in both the alveolar wall and alveolar cavity when compared with the normal control group. However, the abnormality level in mouse lung tissue was somewhat reduced after treatment with IM and TH. The concurrent administration of IM and TH medication considerably enhances the efficacy in ameliorating the abnormality level in mouse lung tissue compared to using either medication individually (Fig. 6F).

The above research findings suggest that synergistically combining the NDM-1 inhibitor TH with IM could effectively restore the sensitivity of CRKP strains to carbapenem antibiotics. This combination notably enhances the survival rate of mice, mitigates the inflammation caused by CRKP infection, and reduces the pathogen load.

#### The combination of IM and SU drugs has a significant therapeutic effect on drug-resistant bacterial infections

**Fig. 7:**
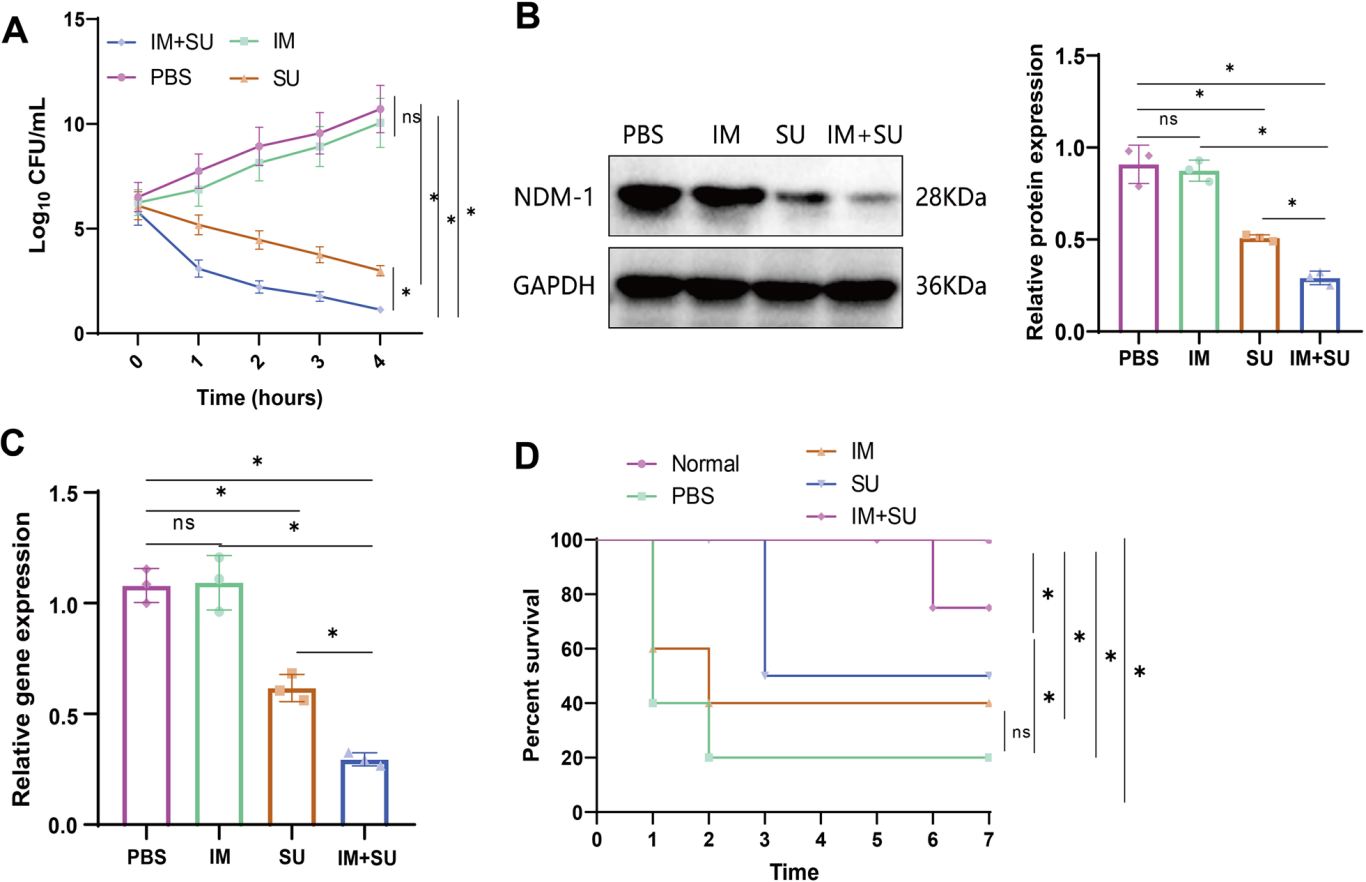
In vitro Inhibition of CRKP Strain Virulence by Combined Treatment with IM and SU. **A** Impact of 24 μM IM, 12 μM SU, and the combination of 24 μM IM and 12 μM SU on the virulence of CRKP strains. **B** Western blot to detect changes in NDM-1 protein expression in CRKP strains after different drug treatments. **C** RT-qPCR to detect changes in blaNDM-1 gene expression in CRKP strains after different drug treatments. **D** Impact of IM, SU, and IM + SU on the survival rate of Galleria mellonella larvae infected with CRKP strains (n = 10). Quantitative data in the figure are represented as mean ± SD, with each experiment repeated 3 times. *P < 0.05 indicates significance between the two groups; ns indicates no difference between the two groups

We conducted a study to assess the impact of the individual and combined administration of IM and SU drugs on CRKP strains. The results indicate that the combination therapy of IM and SU enhanced the ability to inhibit the proliferation of CRKP strains, compared to the IM and SU groups. It is supported by Fig. 7A. The Western blot and RT-qPCR analyses revealed a substantial suppression in the expression of the NDM-1 protein and its associated genes in the CRKP strain following combined treatment with IM and SU (Fig. 7B, C).

Furthermore, the detection results for the survival rate of wax moth larvae revealed that the larvae in the PBS group died after being infected with the CRKP strain, but the mortality rate decreased after treatment. The combination therapy of IM and SU enhanced the larval survival rate during CRKP strain infection compared to their separate use (Fig. 7D).

**Fig. 8:**
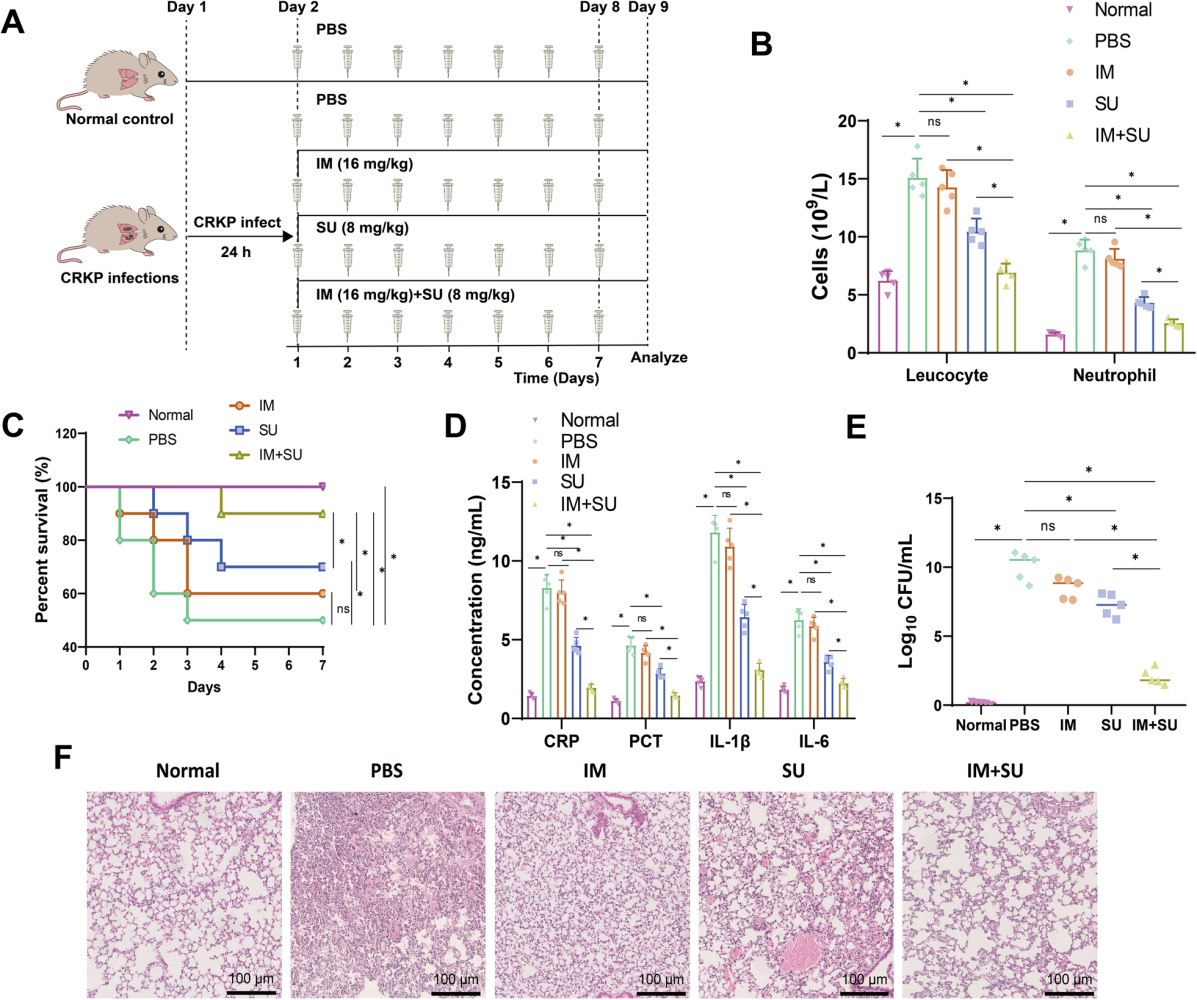
In vivo Inhibition of CRKP Strain Virulence by Combined Treatment with IM and SU. **A** Illustration of mouse drug treatment. **B** Number of white blood cells and neutrophils in mouse blood after 7 days of IM, SU, and IM + SU treatment in CRKP-infected mice (n = 5). **C** IM, SU, and IM + SU impact the survival rate of CRKP-infected mice (n = 10). **D** ELISA to detect the expression of inflammatory factors CRP, PCT, IL-1β, and IL-6 in mouse plasma after 7 days of IM, SU, and IM + SU treatment in CRKP-infected mice (n = 5). **E** Bacterial count in mouse lung tissue after 7 days of IM, SU, and IM + SU treatment in CRKP-infected mice (n = 5). **F** H&E Staining of mouse lung tissue after 7 days of IM, SU, and IM + SU treatment in CRKP-infected mice (scale bar: 200 μm, n = 5). Quantitative data in the figure are represented as mean ± SD, with each experiment repeated 3 times. *P < 0.05 indicates significance between the two groups; ns indicates no difference between the two groups

We conducted additional investigations to examine the therapeutic effects of IM and SU, both individually and in combination, on mice infected with CRKP (Fig. 8A). The blood routine analysis revealed that the infected group of mice exhibited a higher count of white blood cells and neutrophils than the normal group. Compared to the IM group and the SU group, the combined medication group demonstrated a reduction in the number of white blood cells and neutrophils and a decrease in the severity of inflammation (Fig. 8B). The survival status of the mice indicated that all of them perished following infection with CRKP strains. The use of combination therapy enhances mouse survival compared to the individual administration of IM and SU drugs (Fig. 8C).

The detection results of the inflammatory factors PCT, CRP, IL-1β, and IL-6 in the plasma demonstrated a substantial upregulation of PCT, CRP, IL-1β, and IL-6 expression in the infected mice compared to the control group. The combined therapy of IM and SU has a more potent effect in inhibiting the expression of inflammatory factors than individual drug therapy (Fig. 8D).

Colony counting on mouse lung tissue revealed a considerable increase in bacterial numbers in infected mice. Compared to using IM or SU drugs alone (Fig. 8E), the combined administration of IM and SU drugs demonstrates an inhibitory effect on the proliferation of CRKP strains in the body.

Additional histopathological findings from H&E Staining of lung tissue in the infected mice revealed a pronounced infiltration of inflammatory cells and evident damage to the structure of the alveolar walls. Conversely, no abnormalities were detected in the normal control group. Compared to the individual use of the drugs, the combined use of IM and SU drugs improves the severity of abnormalities in mouse lung tissue (Fig. 8F).

To summarize, the combination therapy of the β-lactamase inhibitor ceftazidime/avibactam with IM has the potential to reduce the virulence of CRKP strains, enhance the survival rate of mice, alleviate inflammation caused by CRKP infection, and decrease the pathogen load.

## Discussion

In this study, we conducted an in-depth investigation into the expression regulation of the blaNDM-1 gene in Carbapenem-resistant Gram-negative KP strains from patients with HAP and the optimization of antimicrobial strategies. Prior research indicates significant differences in the expression of the blaNDM-1 gene between resistant and sensitive bacterial strains (Zhang et al. 2023; Wang et al. 2021; Anand et al. 2020). Based on high-throughput sequencing technology, our study found a notable upregulation of blaNDM-1 gene expression in resistant strains compared to sensitive ones. This finding aligns with results reported in the literature, further confirming the critical role of the blaNDM-1 gene in antibiotic-resistant strains.

HAP is a respiratory tract infection that develops in patients during their hospital stay. Patients, often due to underlying diseases, surgical procedures, or prolonged bed rest, experience compromised immune systems, making them more susceptible to infection by pathogens that accumulate in the lungs. The occurrence of HAP poses a serious threat to the patient's life safety as it can lead to severe complications such as sepsis, respiratory failure, and organ dysfunction. This significantly increases the incidence and mortality rate among hospitalized patients. However, the unique hospital environment has led to the gradual development of bacterial resistance to multiple antibiotics. Common examples include methicillin-resistant *Staphylococcus aureus* (MRSA) and multidrug-resistant KP. Infections caused by these drug-resistant strains not only complicate treatment but also result in more severe complications, prolonged hospital stays, and increased mortality rates (Modi and Kovacs 2020). Therefore, investigating the mechanisms underlying the formation of drug-resistant bacteria in HAP and identifying potential therapeutic targets is crucial in providing novel treatment options for HAP patients and potentially saving lives. In our study, we discovered that the expression of the blaNDM-1 gene was significantly upregulated in drug-resistant strains, and we explored the mechanisms by which it contributes to antibiotic resistance. Through in vitro and in vivo experiments, we confirmed that it represents a potential key target for overcoming drug resistance.

In recent years, considerable research has focused on exploring resistance signaling pathways, including the intricate issues related to genomic assembly (Zhang et al. 2022a). Furthermore, studies have demonstrated that various inflammatory signaling pathways lead to complex medical symptoms (Yang et al. 2021b). In contrast to previous studies, our research delved deeper into bioinformatics analysis. We identified resistance-related signaling pathways involving the blaNDM-1 gene and observed enrichment of multiple resistance-related pathways and genomic functions in antibiotic-resistant strains, such as the β-lactam antibiotic degradation pathways. These identified pathways are consistent with previous research findings. However, our study offers a more comprehensive interpretation of their regulation and interactions.

Prior studies have indicated that overexpression of the blaNDM-1 gene leads to enhanced resistance (Wang et al. 2022; Zhang et al. 2022b). Additionally, various reports have underscored the significance of the blaNDM-1 gene in the regulation of antibiotic resistance (Kong et al. 2020; Amabile-Cuevas 2021; Harmer and Hall 2015). In our research, both in vivo and in vitro experiments were conducted to further validate the phenomenon of increased resistance due to the overexpression of the blaNDM-1 gene, aligning with findings reported in the literature. Moreover, in this study, we successfully utilized CRISPR-Cas9 interference technology to downregulate the blaNDM-1 gene. This strategy effectively restored sensitivity in resistant bacterial strains, offering a novel approach to optimizing antimicrobial strategies. These outcomes are congruent with findings from previous research.

Optimized antimicrobial strategies have been documented to enhance survival rates in mice and reduce the pathogen burden (Dolberg et al. 2021). In alignment with these findings, our in vivo experiments aimed at optimizing antimicrobial strategies also markedly improved mouse survival rates and decreased the pathogen load, corroborating previous research outcomes. Rigorous statistical methods were employed to analyze all experimental results, ensuring the reliability and significance of our findings. This statistical analysis further reinforced the scientific robustness and credibility of our study, which is consistent with methodologies used in prior research.

This research work is innovative because it comprehensively explores the relationship between blaNDM-1 gene regulation and drug resistance. Additionally, it introduces a new strategy to optimize antibacterial approaches. Compared to other studies, this research has showcased innovation in research methods, data analysis novelty, and experimental design uniqueness. These innovations offer fresh perspectives on the regulatory mechanisms of drug-resistant bacteria and improve antimicrobial strategies.

## Conclusion

**Fig. 9:**
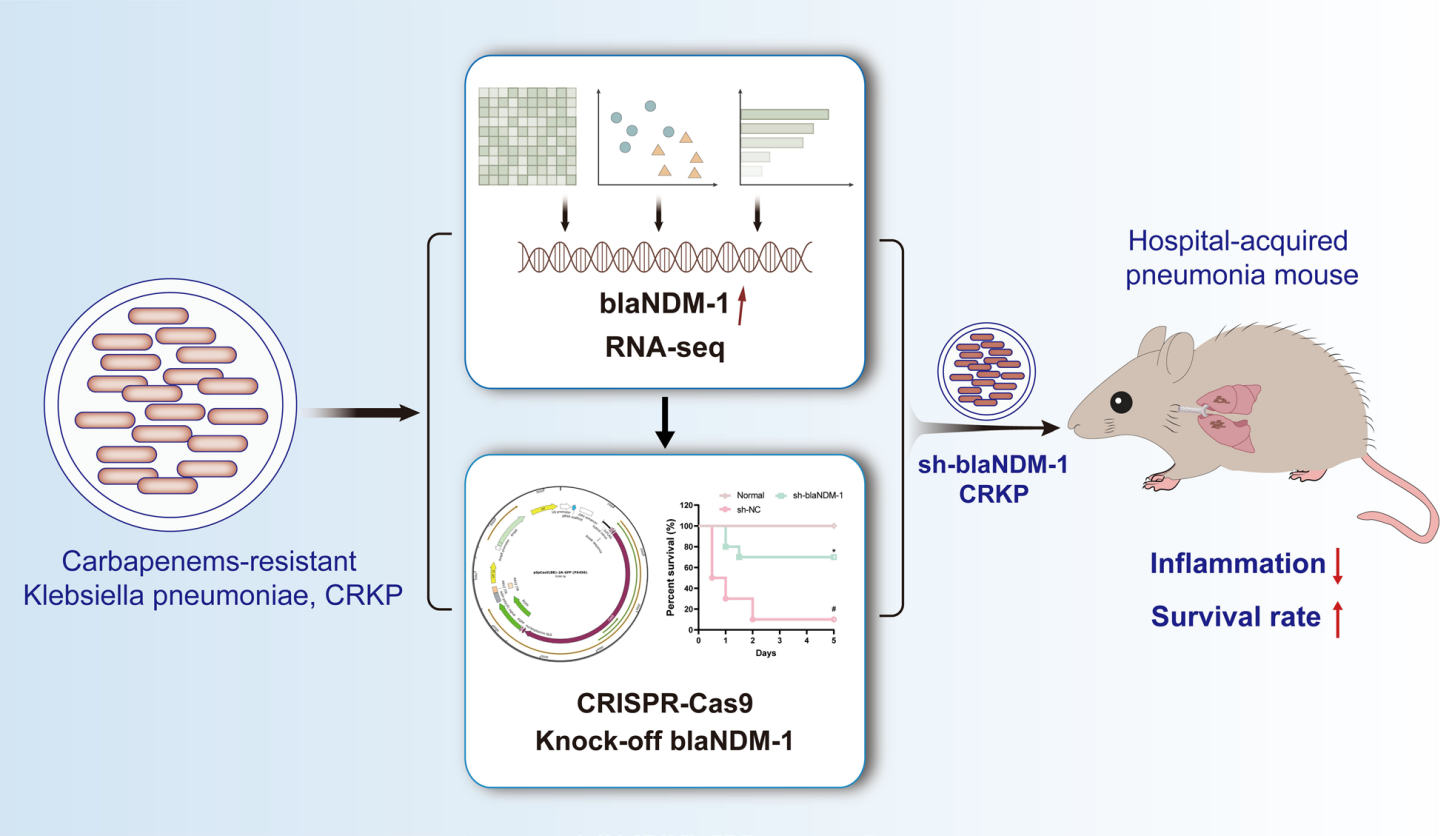
Molecular mechanism of regulation of blaNDM-1 gene on carbapenem resistance in KP and its involvement in the occurrence and development of HAP

In conclusion, the present study offers fresh insights into regulating blaNDM-1 gene expression and optimizing antibacterial strategies. It presents a novel approach for treating HAP-related infections caused by carbapenem-resistant Gram-negative bacteria (Fig. 9). The aforementioned study has made advancements and discoveries in bioinformatics analysis, in vitro experiments, and in vivo animal experiments, surpassing previous research. These findings hold immense importance in tackling the global challenge of antimicrobial resistance.

The present examination investigated the regulation of the blaNDM-1 gene and the optimization of antibacterial strategies in CRKP strains among HAP patients. The research holds significant scientific and clinical value. Firstly, high-throughput sequencing technology has revealed the up-regulation of the blaNDM-1 gene in drug-resistant strains, thereby confirming its role in developing drug resistance. This discovery holds immense importance in elucidating the molecular mechanisms of drug-resistant bacteria. Furthermore, by conducting bioinformatics analysis, we have successfully identified the resistance-related signaling pathway associated with the blaNDM-1 gene. This finding provides crucial insights into the functions and regulatory mechanisms of the blaNDM-1 gene, allowing for a deeper understanding. These findings contribute to the advancement of understanding drug resistance and provide crucial theoretical groundwork for optimizing antibiotic strategies and developing novel drugs.

Furthermore, this study successfully validated in vitro cell and in vivo animal experiments, which demonstrated that the overexpression of the blaNDM-1 Gene causes increased drug resistance. Additionally, interference technology was effectively applied to restore sensitivity in resistant strains. This perspective offers new opportunities to optimize antibacterial strategies and provides alternative treatment options for drug-resistant infections. Optimizing antimicrobial strategies has demonstrated effects in animal experiments, offering valuable guidance for future clinical antimicrobial therapy. These findings are anticipated to pose challenges in tackling drug-resistant bacterial infections in clinical settings. Furthermore, they have the potential to enhance treatment success rates and improve patient survival, thereby offering substantial clinical value.

The present research also has some limitations that need to be addressed. Firstly, the sample size was relatively small, and although we have endeavored to maximize the sample scale, it may still affect the reliability and generalizability of the results. Secondly, this investigation mainly employed in vitro cell experiments and animal experiments for validation, and there might still be differences between the experimental results and the clinical reality, necessitating further verification before clinical application. Additionally, our research focused on carbapenem-resistant Gram-negative bacteria in HAP, and more research support may be needed for the application to other infectious diseases. Although we observed a significant decrease in the resistance of CRKP strains to carbapenem antibiotics when the blaNDM-1 gene was knocked down, the underlying molecular mechanisms have not been experimentally validated. As blaNDM-1 encodes a β-lactamase that is capable of hydrolyzing various β-lactam antibiotics, including penicillins, cephalosporins, and carbapenems, we speculate that the bacteria achieve resistance to multiple antibiotics by upregulating β-lactamase and hydrolyzing the antibiotics. Given the limitations of the current analysis, further investigations are warranted. Firstly, larger sample sizes, including different regions and types of drug-resistant strains, are needed to validate the reliability and generalizability of the results. Secondly, clinical experiments should be further strengthened to explore the prospects of optimizing antimicrobial strategies in clinical treatment. Furthermore, the safety and efficacy of optimizing antimicrobial strategies should be further explored in conjunction with the clinical reality, ensuring the safety and effectiveness of clinical treatment. Simultaneously, experimental validation should be conducted to elucidate the mechanisms by which upregulation of the blaNDM-1 gene promotes the occurrence of resistance.

### Supplementary Information

**Additional file 1: Table S1.** PCR primer sequence. **Table S2.** Western blot antibody information. **Table S3.** RT-qPCR primer sequence.

## Data Availability

The datasets generated and/or analysed during the current study are available in the manuscript and Additional file 1.
