# Supplementary material for: Understanding blaNDM-1 gene regulation in CRKP infections: toward novel antimicrobial strategies for hospital-acquired pneumonia
Source: Mol Med. 2024 Feb 23;30:29. doi: 10.1186/s10020-024-00794-y (PMC10893750; doi:10.1186/s10020-024-00794-y)
Supplement: Supplementary file 1 — Additional file 1: Table S1. PCR primer sequence. Table S2. Western blot antibody information. Table S3. RT-qPCR primer sequence. [file 10020_2024_794_MOESM1_ESM.docx]

**Table S1. PCR** **primer sequence**

| Gene | Primer sequence | Product size (bp) |
| --- | --- | --- |
| IMP | Forward: 5’-CTACGATGATTGCCAGCG-3’ | 399 |
|  | Reverse: 5’-CCATTTGATAATCGCCCTG-3’ |  |
| NDM | Forward: 5’-CACCTCATGTTTGAATTCGCC-3’ | 984 |
|  | Reverse: 5’-CTCTGTCACATCGAAATCGC-3’ |  |
| VIM | Forward: 5’-GATGGTGTTTGGTCGCATA-3’ | 390 |
|  | Reverse: 5’-CGAATGCGCAGCACCAG-3’ |  |

**Table S2. Western blot antibody information**

| Target name | Manufacturer | Item number | Dilution ratio |
| --- | --- | --- | --- |
| NDM-1 (Bacteria) | Novus Biologicals | NBP1-77688 | 1:1000 |
| GAPDH (Bacteria) | Thermofisher | MA5-15738 | 1:1000 |

Note: Antibodies purchased from Thermo Fisher, USA, <https://www.thermofisher.cn/cn/zh/home.html>; or purchased from Novus Biologicals, USA, <https://www.novusbio.com/>

**Table S3. RT-qPCR** **primer sequence**

| Gene | Primer sequence |
| --- | --- |
| blaNDM-1 (KP) | Forward: 5’-CGCAACACAGCCTGACTTT-3’ |
| PMID: 21565805 | Reverse: 5’-TCGATCCCAACGGTGATATT-3’ |
| Probe | Reverse: 5’-6FAM-CAACTTTGGCCCGCTCAAGGTATTT-BHQ1-3’ |
| rho (KP) | Forward: 5’-AACTACGACAAGCCGGAAAA-3’ |
| PMID: 29899556 | Reverse: 5’-ACCGTTACCACGCTCCATAC-3’ |

Note: Primer sequence are cited in PMID: 21565805 and PMID: 29899556. 6FAM: 6-carboxyfluorescein; BHQ1: black hole quencher 1.
